# A Predictive Immunological Signature Associated with Pathological Response in Breast Cancer Treated with Neoadjuvant Chemotherapy

**DOI:** 10.3390/biomedicines14030663

**Published:** 2026-03-14

**Authors:** Luis Arturo Palafox-Mariscal, Mariel García-Chagollán, Jesús García-Gómez, Fabiola Martín-Amaya-Barajas, Valeria Peña-Ruiz, Elizabeth Alvarez-Gonzalez, Eric Alfredo Aranda-Zuno, Jonathan Gallegos-Diaz-de-Leon, Aldo Antonio Alcaraz-Wong, Karina Ordoñez-Pantoja, Raquel Villegas-Pacheco, Adriana Aguilar-Lemarroy, Luis Felipe Jave-Suarez

**Affiliations:** 1 División de Inmunología, Centro de Investigación Biomédica de Occidente (CIBO), Instituto Mexicano del Seguro Social (IMSS), Guadalajara 44340, Jalisco, Mexico; luis.palafox1821@alumnos.udg.mx; 2 Instituto de Investigación en Ciencias Biomédicas, Centro Universitario de Ciencias de la Salud (CUCS), Universidad de Guadalajara, Guadalajara 44340, Jalisco, Mexico; chagollan@academicos.udg.mx; 3 Programa de Doctorado en Ciencias Biomédicas, Centro Universitario de Ciencias de la Salud (CUCS), Universidad de Guadalajara, Guadalajara 44340, Jalisco, Mexico; jesus.garcia9891@alumnos.udg.mx (J.G.-G.); eric.aranda9149@alumnos.udg.mx (E.A.A.-Z.); 4 Programa de Maestría en Ciencias Médicas, Facultad de Medicina, Universidad de Colima, Colima 28040, Colima, Mexico; fabymaya@hotmail.com; 5 Unidad Médica de Alta Especialidad, Hospital de Ginecología y Obstetricia, Centro Médico Nacional de Occidente, Instituto Mexicano del Seguro Social (IMSS), Guadalajara 44349, Jalisco, Mexico; dra.valeriaap@gmail.com (V.P.-R.); lizabed95@outlook.com (E.A.-G.); villegas.raquel@icloud.com (R.V.-P.); 6 Servicio de Patología, Hospital de Especialidades, Centro Médico Nacional de Occidente, Instituto Mexicano del Seguro Social (IMSS), Guadalajara 44349, Jalisco, Mexico; jonathan.adrian.gallegos.diaz@gmail.com (J.G.-D.-d.-L.); draldoalcarazw@hotmail.com (A.A.A.-W.); 7 Laboratorio de Anatomía Patológica y Citologia, Hospital General Regional 110, Instituto Mexicano del Seguro Social (IMSS), Guadalajara 44716, Jalisco, Mexico; karina.ordonez@imss.gob.mx

**Keywords:** breast cancer, immunological signature, pathological response, neoadjuvant chemotherapy

## Abstract

**Background/Objectives**: Breast cancer is a heterogeneous and complex disease with significant individual differences in molecular immunophenotype, biological behavior, histopathological morphology, and response to chemotherapy. The presence of tumor-infiltrating lymphocytes (TILs) has gained considerable attention due to growing evidence of their involvement in therapeutic efficacy, particularly in the response to neoadjuvant chemotherapy (NACT). Different immune cell subsets’ frequency, location, and functional orientation vary substantially between tumor types and individuals with apparently identical cancers. Currently, next-generation sequencing (NGS) has provided key insights into the composition of the tumor microenvironment. Simultaneously, immunohistochemistry (IHC) of paraffin-embedded biopsies allows the visualization of marker proteins within the immune infiltrate, thereby enhancing our understanding of the role of immune cells in cancer therapy. **Methods**: This exploratory study evaluated immune cell tumor infiltration using NGS with immune cell deconvolution, as well as automated IHC on Tru-Cut biopsies from 57 patients with locally advanced breast cancer. Image analysis was performed using Qupath v0.6.0 software. The percentage of infiltrating CD4+ or CD8+ T cells was determined, along with the expression of the markers FoxP3, LAG3, CTLA4, PD1, and TIM-3. We aimed to gain insights into the tumor microenvironment and its influence on the response to NACT in patients with breast cancer. **Results**: Transcriptomic immune deconvolution approaches suggested that a biased cytotoxic tumor environment is linked to chemosensitivity. IHC assays of individual markers reveal that baseline immune cell abundance and individual checkpoint expression did not differ significantly across the response groups. However, the functional organization and coordination of the tumor immune microenvironment showed distinct associations with chemosensitivity. **Conclusions**: Features representing immune balance, such as CD8/CD4 ratio and T cell-contextualized metrics, emerged as candidate predictors of pathological response to NACT, outperforming molecular phenotype alone in this exploratory cohort.

## 1. Introduction

Breast cancer remains the most frequently diagnosed cancer among women and a leading cause of cancer-related mortality worldwide. According to the World Health Organization, over 2.3 million women were diagnosed with breast cancer globally in 2020 [1]. This disease is characterized by the uncontrolled proliferation of cells in breast tissue, mainly in the ducts (ductal carcinoma) or in the lobules (lobular carcinoma) [2]; once established, it has an affinity for spreading through the lymphatic system (mainly to the axillary lymph nodes) and through the bloodstream to distant organs such as bones, lungs, liver, or brain [3,4,5]. Far from being a single entity, breast cancer encompasses a spectrum of diseases with marked heterogeneity, which complicates its therapeutic management. While this variability is well documented by molecular and genetic studies, clinical practice often relies on a surrogate molecular profiling for reasons of accessibility and cost-effectiveness. This approach uses standard immunohistochemistry (IHC) to evaluate the expression of four key markers—estrogen receptors (ERs), progesterone receptors (PRs), HER2, and the Ki-67 index—to estimate the ‘intrinsic subtype’ of the cancer. These markers distinguish four main subtypes: Luminal A (ER+, PR+, HER2-, low Ki-67), Luminal B (ER+, PR±, HER2±, high Ki-67), HER2+ (ER-, PR-, HER2+), and triple-negative (ER-, PR-, HER2-) [5,6,7].

Despite advances in early detection and therapy, disparities in outcomes persist due to tumor heterogeneity, late-stage diagnosis, and resistance to conventional treatments [8]. The breast cancer treatment is multimodal and includes surgery along with systemic therapy. The latter can be administered prior to surgery to reduce tumor burden, a strategy known as neoadjuvant chemotherapy (NACT). When administered after surgery to eliminate any residual disease, it is called adjuvant therapy [9,10]. NACT is the standard treatment for locally advanced or inflammatory breast cancer and is also used in early-stage disease. It is widely employed to downstage tumors, improve surgical outcomes, and provide early insights into treatment response [11]. Its efficacy is determined by assessing the pathological response, a measure of tumor reduction that serves as a crucial prognostic indicator [11]. A key goal is to achieve a pathological complete response (pCR), defined as the disappearance of all invasive tumor cells in the breast and lymph nodes after NACT. The likelihood of achieving a pCR varies significantly depending on the molecular subtype of the breast tumor. For example, triple-negative tumors exhibit a good response to NACT compared to luminal tumors [12]. However, pCR rates are disappointing, with less than 31% of patients achieving a treatment-sensitive response or pCR. Many patients who initially respond well to this therapy later develop recurrences, often linked to acquired resistance to chemotherapeutic agents [13]. Resistance to NACT poses a major clinical challenge; therefore, understanding the biological mechanisms behind its development is crucial for improving patient outcomes. Such resistance not only limits therapeutic options but also reduces the 5-year survival rate, highlighting the influence of intrinsic factors on treatment response [14,15].

Recent evidence has highlighted the role of the tumor microenvironment (TME) in modulating therapeutic response. Immune cells within the TME, including tumor-associated macrophages, T cells, and natural killer (NK) cells, can either support or hinder anti-tumor activity [16,17,18]. Tumor-infiltrating lymphocytes (TILs) are key components of the TME and reflect the immune response against cancer. In the context of NACT for breast cancer, TILs have emerged as predictive biomarkers of treatment response, especially in aggressive subtypes such as triple-negative breast cancer (TNBC) and HER2+ [19,20,21]. The presence of TILs reflects a pre-existing anti-tumor immune response, which often correlates with better long-term clinical outcomes after chemotherapy. Consequently, TILs are considered an independent biomarker of better disease-free survival in breast cancer patients undergoing NACT [22,23]. Studies suggest that high levels of intratumoral and stromal TILs are associated with a higher likelihood of achieving a pCR [24]. For instance, TNBC tumors with TILs greater than 30% have shown higher pCR rates [25,26]. In HER2+ tumors, the presence of TILs improves the efficacy of anti-HER2 therapies (such as trastuzumab) when combined with NACT [27]. In the KEYNOTE-522 study, Schmid et al. observed that patients with TNBC and high TILs showed a higher pCR rate when pembrolizumab (an anti-PD1 agent) was added to NACT, reinforcing the critical role of the immune microenvironment [28]. However, the tumor infiltrate presents spatial, temporal, and cellular heterogeneity. Therefore, its evaluation requires precise quantification methods and markers that indicate specific immune phenotypes. The specific determination of the lymphocyte phenotypes within the tumor infiltrate may be an essential indicator of the immune response to NACT. Integrating this information as a diagnostic or prognostic biomarker could provide predictive models for NACT response. This would enable personalized therapeutic strategies and optimized patient selection for combination therapies.

In the present study, we quantified the percentage of infiltrating CD4+ or CD8+ T cells, as well as the expression of FoxP3, LAG3, CTLA4, PD1, and TIM-3 in tumor tissue of patients with breast cancer who were candidates to NACT. We aimed to gain insights into the tumor microenvironment and its influence on the response to NACT.

## 2. Materials and Methods

### 2.1. RNAseq Analysis and Immune Deconvolution Algorithms

For RNAseq analysis and immune deconvolution, data from our group’s prior study (GSE162187) were used. In that study, biopsies from breast cancer patients pre-NACT administration were sequenced by NGS. Pathological response to neoadjuvant chemotherapy was evaluated using the Residual Cancer Burden (RCB) index. Response categories include RCB 0 (pCR), RCB I (minimal residual disease), RCB II (moderate), and RCB III (extensive) [15,29]. For analytical purposes, tumors achieving a pathological complete response (RCB 0) and minimal residual disease (RCB I) were classified as chemosensitive, whereas tumors with substantial residual disease (RCB II–III) were classified as chemoresistant.

The raw RNAseq data were analyzed de novo. The sequencing quality was assessed by FastQC, and sequence alignment was performed using RSubread version 2.24.0. FeatureCounts of RSubread package was used to generate tables with transcripts abundance. Immune cell infiltration was determined in all samples by deconvolution algorithms. This analysis was conducted using the omnideconv/immunedeconv package (https://github.com/omnideconv/immunedeconv; last accession 12 January 2026) within the R platform version 4.5.2. Specifically, the quanTIseq, xCell, and EPIC algorithms were applied.

### 2.2. Breast Cancer Patients

Clinical data and Tru-Cut Paraffin-embedded biopsies were obtained from 57 breast cancer patients scheduled for NACT from 2020 to 2022. All samples were provided by the Unidad Médica de Alta Especialidad (UMAE) of the Centro Médico Nacional de Occidente and the Hospital General Regional 110 Oblatos of the Instituto Mexicano del Seguro Social (IMSS) in Guadalajara, Jalisco, Mexico. Biopsies were collected prior to the initiation of any treatment. For inclusion, these biopsies were previously fixed in 10% formaldehyde and subsequently included in paraffin, this was performed by the UMAE pathology services. Tumor molecular phenotype was determined using an immunohistochemistry-based surrogate classification according to the St. Gallen 2011 consensus, based on estrogen receptor (ER), progesterone receptor (PR), HER2, and Ki-67 expression. HER2-positive tumors were defined as ER- and PR-negative with HER2 overexpression, while ER- and/or PR-positive, HER2-positive tumors were classified as Luminal B HER2-positive and analyzed within the Luminal B group.

### 2.3. Construction of Receptors Blocks for Tissue Array

To generate the receptors blocks for the tissue array, a custom mold was designed and 3D-printed using Superflex 80A flexible resin (Cat. No. B08RWMMVCH, 3D Materials, Anyang-si, Republic of Korea). The mold contained 11 columns, each 50 mm in height and 4 mm in diameter, to generate the wells of the receptor block. To fabricate a block, 10 g of paraffin wax (Surgipath Paraplast, Cat. No. 39601006, Leica Biosystems, Wetzlar, Germany) was melted at 60 °C, poured into the mold, quickly placed a biopsy block cassette (Leica LP C Biopsy Cassettes, Cat. No. 14060546833, Leica Biosystems, Wetzlar, Germany), and left to dry for 2 h, after which the mold was removed, leaving the receptor block ready for use.

### 2.4. Tissue Arrays

For each Tru-Cut biopsy, a hematoxylin–eosin (H&E) slide was evaluated to identify the tumor region. Subsequently, a 4 mm tissue core was extracted from the block using a disposable dermal punch (Disposable Biopsy/Dermal Punch-4mm, Cat. No. TOP001-4mm, Miltex (Integra LifeSciences), York, PA, USA) and inserted into a designated well of the receptor block. A total of nine biopsies from different patients were placed per receptor block, a tonsillar sample with tonsillitis was added as a positive control tissue, and one well was left empty to easily locate the orientation after sectioning. To align the samples, the receptor block was placed on a glass slide on a heating plate at 45 °C for 5 s to melt only the surface and secure the samples in place. The block was then cooled to room temperature, and the glass slide was carefully removed. Sections of 4 μm thickness were cut from each tissue array using a microtome (Cat. No. RM2125 RTS, Leica Biosystems, Wetzlar, Germany) with a 5° clearance angle. The sections were then placed in a distilled water bath at 43 °C, recovered on electrocharged slides (Apex Adhesive Slide, Cat. No. 3800080E, Leica Biosystems, Wetzlar, Germany), dried in an oven for 1 h at 40 °C, and subsequently left to air-dry at room temperature overnight.

### 2.5. Immunohistochemistry Assays

Immunohistochemistry (IHC) was performed on the Bond Max automated stainer (Cat. No. 49.0051, Leica Biosystems, Wetzlar, Germany) following the manufacturer’s protocol. The slides were placed in the equipment trays and covered with Leica covertiles (Cat. No. S21.4611, Leica Biosystems, Wetzlar, Germany). The primary antibodies and dilutions used were Anti-CD4 1:200 ([MSVA-004R] HistoMAX™, Cat. No. GTX04379, GeneTex, Irvine, CA, USA); anti-CD8 1:400 ([LT8], Cat. No. GTX74778, GeneTex, Irvine, CA, USA); anti-PD1 1:200 ([NAT105], Cat. No. GTX20256, GeneTex, Irvine, CA, USA); anti-FoxP3 1:200 (D2W8E™, Cat. No. 98377, Cell Signaling, MA, USA); anti-LAG3 1:200 (D2G4O XP^®^, Cat. No.15372, Cell Signaling, Danvers, MA, USA); anti-CTLA4 1:200 (E2V1Z, Cat. No. 53560, Cell Signaling, Danvers, MA, USA); and anti-TIM-3 1:100 (D5D5R™ XP^®^, Cat No. #45208, Cell Signaling, MA, USA). For all antibodies, heat-induced epitope retrieval (HIER) was performed at 100 °C for 20 min using Bond Epitope Retrieval Solution 2 (ER2, EDTA-based buffer, pH 9, Cat. No. AR9640, Leica Biosystems, Wetzlar, Germany). Primary antibodies were incubated at room temperature for 15 min. Detection was performed using the BOND Polymer Refine Detection system (Cat. No. DS9800, Leica Biosystems, Wetzlar, Germany). After the automated process, slides were manually dehydrated through graded ethanol (95% and absolute) and xylene (two immersions of 15 s each). Finally, the slides were mounted using CV Ultra Mounting Medium (Cat. No. 14070937891, Leica Biosystems, Wetzlar, Germany), coverslipped, and dried overnight at room temperature.

### 2.6. Analysis of IHC Images

To analyze the percentage of positive cells for each marker, four representative microphotographs were captured per tissue at 10X magnification using a Leica DM500 optical microscope coupled to an ICC50E camera (Leica Biosystems, Wetzlar, Germany). Images were saved in JPG format (2 MP, 1600 × 1200) (Supplemental Figures S1–S7) and subsequently analyzed without knowledge of the RCB results, molecular subtype, grade, or any other clinical data of the patients evaluated to avoid bias during the process. To minimize variability in hematoxylin and Diaminobenzidine (DAB) staining intensity, Macenko normalization was applied using a reference image for each marker in which the hematoxylin signal and the positive DAB signal were clearly distinct [30,31]. Macenko normalization was perform as described in https://www.geeksforgeeks.org/machine-learning/macenko-method-for-normalizing-histology-slides-for-quantitative-analysis/ (last accession 28 February 2026). Normalization was performed using Python software version 3.13.9.

Normalized images were subsequently analyzed using QuPath version 0.6.0 [32]. For this purpose, separate projects were created for each antibody, and normalized images were imported. For staining vectors estimation, a reference area in the normalized reference image was selected. Staining vectors were calibrated using the ‘auto’ command, and a script was generated through the workflow manager to standardize these vectors across all images in the project using the ‘automate’ tool. This step ensured accurate color deconvolution [32].

For positivity analysis, a rectangular region of interest (ROI) covering the entire image was defined. Cell detection was performed by applying parameters optimized for lymphocytes, as described by Berben et al. (2020) [33]. Under pathologist supervision, a detection script was generated and batch processed. After verifying the accuracy of lymphocyte detection, the percentage of positive cells was determined using the ‘Positive Cell Detection’ tool. The following intensity threshold values were established for each marker: CD4 (0.25), CD8 (0.42), CTLA4 (0.25), LAG3 (0.22), PD1 (0.26), TIM-3 (0.60), and FoxP3 (0.45). These intensity threshold values were chosen through manual calibration under the supervision of a pathologist, ensuring that only lymphocytes visually positive for DAB staining were marked as positive. Subsequently, these parameters were automated via scripting and applied to all project images. Following the analysis, each image was reviewed to remove artifacts generated by the IHC process; images with significant tissue damage or loss that precluded adequate analysis were discarded. For each evaluated marker, the average percentage of positive cells per patient was calculated by averaging the results of all tissue sections analyzed from at least three images. The results were evaluated by the software operator and validated by the pathologist. (Scripts for Macenko normalization and cell quantification using QuPath 0.6.0 are available in the Supplementary GitHub repository: https://github.com/Luis-Arturo-Palafox/Scripts-for-image-analysis- (accessed on 4 March 2026).

### 2.7. Statistical Analysis

All statistical analysis were performed using Python, version 3.13, with standard scientific libraries including scikit-learn (version 1.5.2), pandas (version 2.2.3), NumPy (version 2.1.0), SciPy (version 1.14.1), Matplotlib (version 3.9.2), and Seaborn (version 0.13.2.). For visualization purposes, a log2 scale was applied to y-axes in plots to better represent data distribution, while untransformed data were preserved and used for statistical testing. For the immune cell deconvolution analysis, differences in cell population enrichment were evaluated using two-sided Mann–Whitney U tests. Effect sizes were quantified using the rank biserial correlation coefficient, where positive values indicate higher enrichment (r) in chemoresistant samples. Differences in IHC marker positivity across breast cancer phenotypes and RCB classes were assessed using the Kruskal–Wallis H test with eta-squared (η^2^) effect sizes. Post hoc pairwise comparisons were conducted using Mann–Whitney U tests. Spearman’s rank correlation coefficients were calculated for all pairwise combinations of IHC markers, stratified by RCB class. *p*-values from Mann–Whitney U tests, Kruskal–Wallis tests, and Spearman correlation analysis were adjusted for multiple testing using the Benjamini–Hochberg false discovery rate (FDR) correction method. For correlation analysis, FDR correction was applied separately within each RCB class.

To assess the stability of correlation estimates, particularly in smaller RCB strata, bootstrap confidence intervals were calculated for all Spearman correlations. For each correlation within each RCB group, 1000 bootstrap resamples with replacement were generated, and the 2.5th and 97.5th percentiles of the resulting distribution provided 95% confidence intervals.

Systematic feature selection for the Random Forest model from seven IHC markers (CD4, CD8, LAG3, CTLA4, FOXP3, PD1 and TIM-3) was performed. A total of 80 candidate features were generated across four categories: single markers, pairwise products, pairwise ratios, and T cell-contextualized features ([CD8 × Regulatory]/CD4 and [CD4 × Regulatory]/CD8) for each of the five immune regulatory markers (PD1, CTLA4, LAG3, TIM-3, FOXP3). For all analyses, pathological complete response (pCR; RCB 0, *n* = 20) was encoded as the negative class (0) and residual disease (RD; RCB I–III, *n* = 37) as the positive class (1). Each feature’s discriminatory capacity for pCR against RD was evaluated using receiver operating characteristic (ROC) curve analysis. Optimal thresholds were determined by Youden’s index, and statistical significance was assessed by Fisher’s exact test. Odds ratios with 95% confidence intervals were calculated. Features were ranked by area under the curve (AUC), and the Benjamini–Hochberg FDR correction was applied to the *p*-value for the top five features. These features, along with the phenotype encoded as dummy variables, were selected for the design of a Random Forest classifier for the discrimination of pCR (*n* = 20) vs. RD (*n* = 37). The model was configured with 100 trees with balanced class weights and evaluated using a 5-fold stratified cross-validation. Out-of-fold (OOF) predictions were poled across fold to derive a single pooled OOF AUC. A 95% confidence interval for the pooled OOF AUC was estimated using bootstrap resampling of the OOF predictions at 1000 iterations. To validate that the model’s performance exceeded chance expectations, a one-sided Monte Carlo permutation test was conducted with 1000 random permutations of class labels. To quantify the incremental value of immune features, a baseline clinical-only model was trained using the phenotype data alone under identical cross-validation conditions. Comparison between models was characterized by three complementary metrics: the difference in pooled OOF AUC (point estimate Δ), and the differences in mean and median of the bootstrap AUC distributions (Δ mean bootstrap, Δ median bootstrap), along with 95% confidence intervals for both models. Cross-validated metrics included accuracy, sensitivity, specificity, positive predictive value (PPV), negative predictive value (NPV), and ROC-AUC with 95% confidence intervals. Feature importance was quantified by mean decrease in Gini impurity. For all tests, a two-sided alpha of 0.05 was used to establish statistical significance.

## 3. Results

### 3.1. Immune Deconvolution Algorithms Highlight Differences in T Cell Populations in Patients with Different NACT Responses

Advances in transcriptomic technologies, particularly bulk RNA sequencing (RNAseq) coupled with immune deconvolution algorithms, have enabled the characterization of the immune landscape in tumor samples at unprecedented resolution. Immune deconvolution algorithms are computational methods that use gene expression data to estimate the proportions of different immune cell types within a sample. These algorithms work by comparing the gene expression patterns of a sample to pre-defined gene signatures of known immune cell types [34]. In a previous study, our research group evaluated transcriptomic information from patients who were sensitive and resistant to NACT using RNAseq (GSE162187) [35]. After reviewing the data obtained in that study, we wondered what the immunological landscape associated with treatment response would be. To answer our question, we applied the xCell deconvolution algorithm to this dataset to estimate the proportions of immune cell populations in both groups.

**Figure 1:**
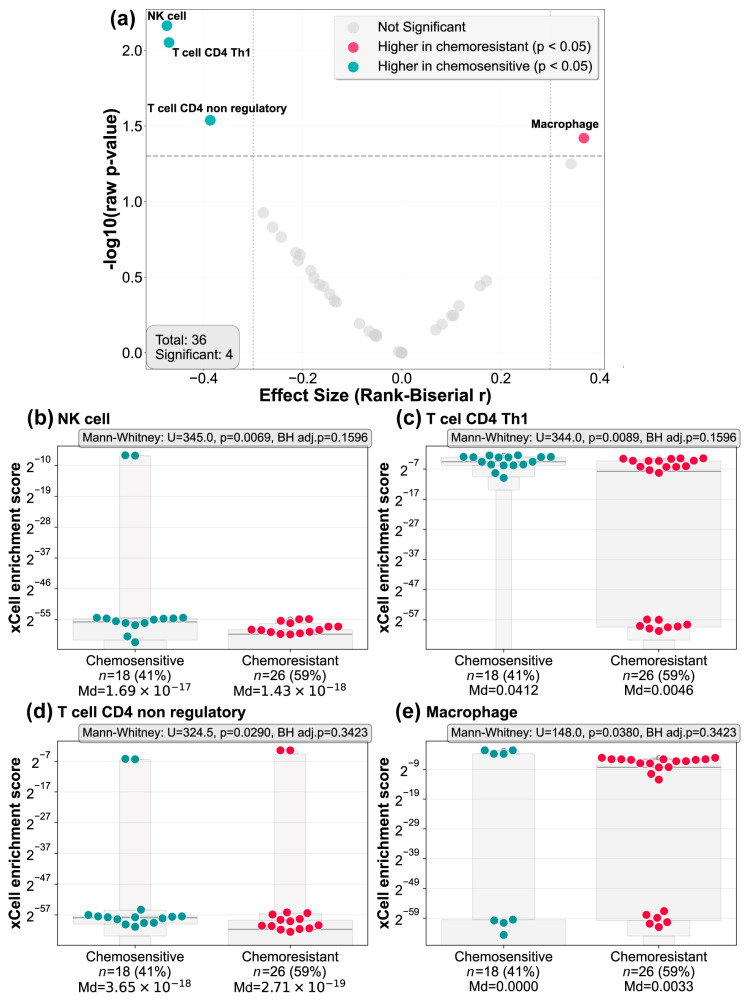
Immune deconvolution analysis reveals differential immune cell enrichment. (**a**) Volcano plot showing the effect size (Rank Biserial r) and significance (−log10 raw *p*-value) of immune cell populations inferred by the xCell algorithm comparing chemosensitive and chemoresistant tumors. Each point represents a distinct cell type. Green points indicate cell types nominally enriched in chemosensitive tumors, pink points represent those enriched in chemoresistant tumors, and gray points represent non-enriched lineages. Dashed lines indicate significance thresholds (*p* = 0.05, horizontal; r = −0.3 and r = +0.3, vertical). Natural killer (NK) cells, CD4+ Th1 T cells, and CD4+ non-regulatory T cells were nominally elevated in chemosensitive samples, while macrophages were enriched in chemoresistant samples. Box plots depicting xCell enrichment scores (log_2_ space with untransformed y-axis tick labels) for immune cell populations showing nominal differences between groups: (**b**) NK cells, (**c**) CD4+ Th1 T cells, (**d**) CD4+ non-regulatory T cells, and (**e**) macrophages. Each point represents an individual patient sample. The gray enhanced box plot contours display letter-value summaries, with box widths proportional to the square root of the number of observations at each level. Each panel displays raw median scores and Mann–Whitney U test statistics with both uncorrected and Benjamini–Hochberg-adjusted *p*-values (BH adj. *p*) performed in untransformed data. Chemosensitive tumors displayed higher nominal enrichment of NK cells and CD4+ T cell subsets, whereas chemoresistant tumors exhibited nominally increased macrophage enrichment. These findings, while exploratory, prompted targeted immunohistochemical (IHC) investigation of T cell subsets and checkpoint molecules.

Volcano plot analysis comparing effect sizes and statistical significance revealed four immune populations with nominal enrichment between the response groups (Figure 1a). To visualize the full distribution of effect sizes across all 36 tested cell lineages, the volcano plot displays uncorrected *p*-values, while Benjamini–Hochberg-adjusted *p*-values are reported in individual box plots (Figure 1b–e) for full transparency. Specifically, tumors from chemosensitive patients showed nominally significant enrichment in natural killer (NK) cells (*p* = 0.0069, BH adj. *p* = 0.1596), CD4+ Th1 T cells (*p* = 0.0089, BH adj. *p* = 0.1596), and CD4+ non-regulatory T cells (*p* = 0.0290, BH adj. *p* = 0.3423). In contrast, chemoresistant tumors exhibited enrichment of macrophages (*p* = 0.0380, BH adj. *p* = 0.3423). While none of these signals survived FDR correction in this limited cohort (*n* = 44), the pattern of enhanced cytotoxic immune cell populations in chemosensitive tumors aligns with established mechanisms of chemotherapy-induced immunogenic cell death and prompted further investigation via targeted immunohistochemistry.

### 3.2. Distribution of the Immunological Markers According to Tumor Phenotype

**Figure 2:**
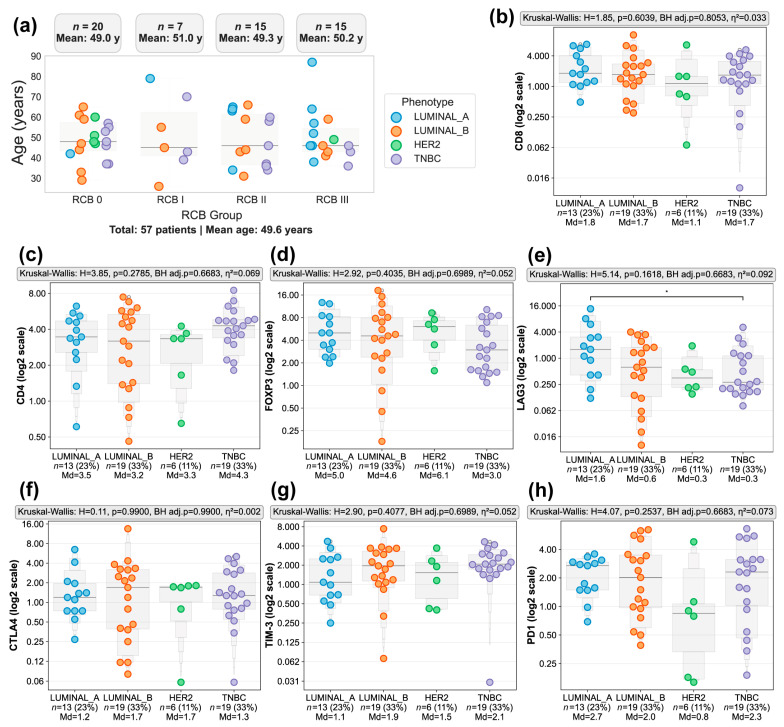
Immunohistochemical characterization of immune markers across breast cancer phenotypes in patients treated with neoadjuvant chemotherapy (NACT). (**a**) Distribution of patient age across Residual Cancer Burden (RCB) classes (RCB 0–III). Each point represents an individual patient, colored by tumor phenotype (Luminal A *n* = 13, Luminal B *n* = 19, HER2+ *n* = 6, or triple-negative breast cancer [TNBC] *n* = 19). Mean age for each RCB class is shown. Expression of immune markers across breast cancer phenotypes evaluated by IHC are displayed in a log_2_ space with untransformed y-axis ticks: (**b**) CD8, (**c**) CD4, (**d**) FOXP3, (**e**) LAG3, (**f**) CTLA-4, (**g**) TIM-3, and (**h**) PD1. Gray enhanced box plot contours display letter-value summaries, with box widths proportional to the square root of the number of observations at each level. Panels display raw median values (shown below each phenotype) and statistical test results. Statistical comparisons were performed on untransformed data using the Kruskal–Wallis test; H values, uncorrected *p*-values, Benjamini–Hochberg-adjusted *p*-values (BH adj. *p*), and effect sizes (η^2^) are shown above each panel. An asterisk (*) indicates significant pairwise comparison (Mann–Whitney U test, *p* < 0.05).

To characterize the immune landscape associated with the response to NACT at the protein level, we analyzed immunohistochemical markers in 57 breast cancer patients with a mean age of 49.6 years, categorized by tumor molecular phenotype and RCB (Table S1). The cohort comprised Luminal A (*n* = 13, 22.8%), Luminal B (*n* = 19, 33.3%), HER2-enriched (*n* = 6, 10.5%), and TNBC (*n* = 19, 33.3%) phenotypes. Figure 2a illustrates the distribution of molecular phenotypes across the four RCB classes (RCB 0–III). Age was similar across RCB groups (mean ranges: 49.3–51.0 years), and the distribution of tumor phenotypes showed a clear pattern related to treatment response.

Luminal A tumors were overrepresented in RCB II and RCB III, revealing that this phenotype tended to have higher residual disease and poorer response to NACT. In contrast, HER2+, Luminal B and TNBC phenotypes were predominant in RCB 0 (pathological complete response or pCR), but showed a more heterogeneous distribution, spanning multiple RCB categories. Of note, among the six HER2+ patients, five achieved RCB 0 response while only one had RCB III.

After characterizing these phenotype-response patterns, we evaluated whether differences in immune marker expression could explain the observed variability in treatment outcomes. Across the four molecular phenotypes (Luminal A, Luminal B, HER2+, and TNBC), no statistically significant differences were found for any of the immune markers assessed by IHC, nominally or after Benjamini–Hochberg correction. CD8 expression showed comparable levels across groups (*p* = 0.6039, BH adj. *p* = 0.8053, η^2^ = 0.033; Figure 2b), with median values ranging from 1.1 to 1.8. Similarly, CD4 expression (*p* = 0.2785, BH adj. *p* = 0.6683, η^2^ = 0.069; Figure 2c) and FOXP3 cell positivity (*p* = 0.4035, BH adj. *p* = 0.6989, η^2^ = 0.052; Figure 2d) showed no significant differences between phenotypes, although TNBC and Luminal B tumors displayed slightly higher median values. However, the limited HER2-enriched representation precludes definitive conclusions about this subtype.

Regarding the expression of immune checkpoints molecules, LAG3 showed variable expression across phenotypes, with a numerical difference between Luminal A (median 1.6%) and TNBC (median 0.3%) (Figure 2e). In contrast PD1 (*p* = 0.2537, BH adj. *p* = 0.6683 η^2^ = 0.073; Figure 2h), CTLA-4 (*p* = 0.9900, BH adj. *p* = 0.9900 η^2^ = 0.002; Figure 2f), and TIM-3 (*p* = 0.4077, BH adj. *p* = 0.6988 η^2^ = 0.052; Figure 2g) showed no statistically significant differences among phenotypes. However, it is noteworthy that some markers exhibited wide dynamic ranges, particularly PD1 and CTLA-4, whose expression values spanned from near-zero to more than 4% positivity. This variability was most evident in Luminal B and TNBC tumors, which also showed heterogeneous RCB responses. This suggests that baseline checkpoint heterogeneity may reflect underlying immunological diversity that contributes to treatment outcomes beyond phenotype alone in our exploratory cohort.

### 3.3. T Cell Infiltration Across RCB Response Groups

**Figure 3:**
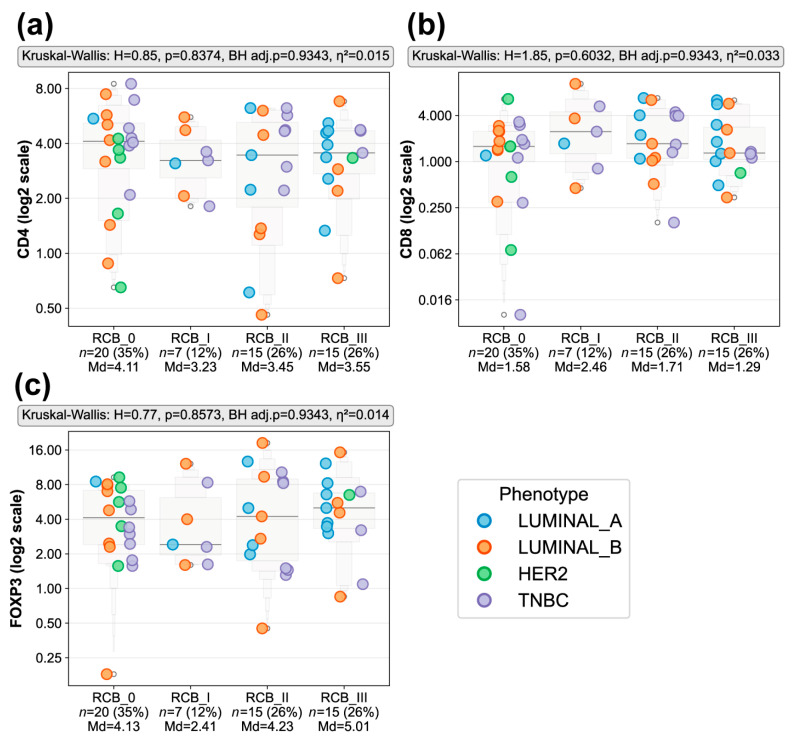
Baseline T cell infiltration across RCB response groups. Immunohistochemical quantification of (**a**) CD4, (**b**) CD8, and (**c**) FOXP3 expression across RCB classes (RCB 0 *n* = 20, RCB I *n* = 7, RCB II *n* = 15, RCBIII *n* = 15). Each dot represents an individual tumor sample and is colored according to molecular phenotypes (Luminal A, Luminal B, HER2+, and TNBC). Gray enhanced box plot contours display letter-value summaries, with box widths proportional to the square root of the number of observations at each level. Expression values are shown on a log_2_ spaced y-axis, with untransformed tick labels and raw median values displayed below each RCB group. Statistical comparisons among RCB groups were performed on untransformed data using the Kruskal–Wallis test; corresponding H statistics, uncorrected *p*-values, Benjamini–Hochberg-adjusted *p*-values, and effect sizes (η^2^) are displayed above each panel.

To determine whether baseline T cell infiltration differed according to pathological response to NACT, we quantified CD4, CD8, and FOXP3 expression by IHC across RCB classes (RCB 0–III). The cohort distribution by pathological response was RCB 0 (*n* = 20, 35.1%), RCB I (*n* = 7, 12.3%), RCB II (*n* = 15, 26.3%), and RCB III (*n* = 15, 26.3%). As shown in Figure 3, no statistically significant differences were detected in the expression of CD4 (*p* = 0.8374, BH adj. *p* = 0.9343, η^2^ = 0.015), CD8 (*p* = 0.6032, BH adj. *p* = 0.9343, η^2^ = 0.033), or FOXP3 (*p* = 0.8573, BH adj. *p* = 0.9343, η^2^ = 0.014) across the four RCB groups. Median expression values for each marker were comparable between chemosensitive (RCB 0–I) and chemoresistant (RCB II–III) tumors, suggesting that baseline T cell infiltration did not differ significantly by degree of residual disease in this exploratory cohort.

Although not statistically significant, CD4 and FOXP3 expressions tended to show greater dispersion in RCB III tumors, whereas CD8 levels were slightly higher in RCB I. However, the limited sample size, particularly for RCB I (*n* = 7), precludes definitive interpretation of these trends.

Given that baseline T cell infiltration, as assessed by CD4, CD8, and FOXP3 expression, did not differ significantly across RCB response categories, we next evaluated whether functional markers of T cell regulation and exhaustion were associated with pathological response to NACT. We assessed the expression of the immune checkpoint molecules LAG3, PD1, CTLA-4, and TIM-3 across RCB classes (RCB 0–III).

**Figure 4:**
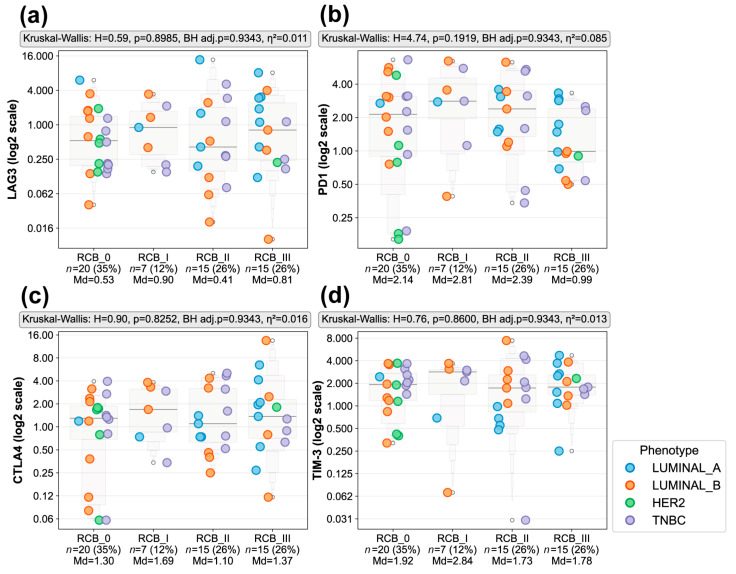
Immune checkpoint expression across RCB categories in pre-treatment biopsies. Expression of (**a**) LAG3, (**b**) PD1, (**c**) CTLA-4, and (**d**) TIM-3 was assessed by IHC in pre-treatment biopsies and stratified according to pathological response to NACT, classified as RCB 0–III (RCB 0 *n* = 20, RCB I *n* = 7, RCB II *n* = 15, RCB III *n* = 15). Each dot represents an individual tumor sample, colored by molecular phenotypes (Luminal A, Luminal B, HER-2+, or TNBC). Gray enhanced box plot contours display letter-value summaries, with box widths proportional to the square root of the number of observations at each level. Positivity percentages are displayed on a log2 spaced y-axis with untransformed tick labels; raw median values are shown below each RCB group. Statistical comparisons among RCB groups were performed on untransformed data using the Kruskal–Wallis test; H statistics, uncorrected *p*-values, Benjamini–Hochberg-adjusted *p*-values (BH adj. *p*), and effect sizes (η^2^) are displayed above each panel. No significant differences in immune checkpoint expression were observed across RCB categories.

As shown in Figure 4, no statistically significant differences were observed in the expression of LAG3 (*p* = 0.8985, BH adj. *p* = 0.9343, η^2^ = 0.011), PD1 (*p* = 0.1919, BH adj. *p* = 0.9343, η^2^ = 0.085), CTLA-4 (*p* = 0.8252, BH adj. *p* = 0.9343, η^2^ = 0.016), or TIM-3 (*p* = 0.8600, BH adj. *p* = 0.9343, η^2^ = 0.013) among RCB groups. Median expression levels for each checkpoint marker were comparable between chemosensitive (RCB 0–I) and chemoresistant (RCB II–III) tumors, with small effect sizes (η^2^ = 0.011–0.085), suggesting that checkpoint expression alone did not differ by RCB in this cohort.

Despite the lack of statistical significance, checkpoint makers, particularly PD1 and CTLA-4, displayed a broad dynamic range of expression across all RCB categories, spanning from low to high positivity percentages (Figure 4). This variability was observed within both chemosensitive and chemoresistant tumors and was independent of molecular phenotype distribution. The heterogeneous expression of immune checkpoint molecules highlights the importance of assessing T cell functional states, in addition to cell abundance alone, to better understand the immunologic mechanisms underlying sensitivity or resistance to NACT.

### 3.4. Correlations Between Immunological Markers and Immune Checkpoints Differ According to Pathological Response

**Figure 5:**
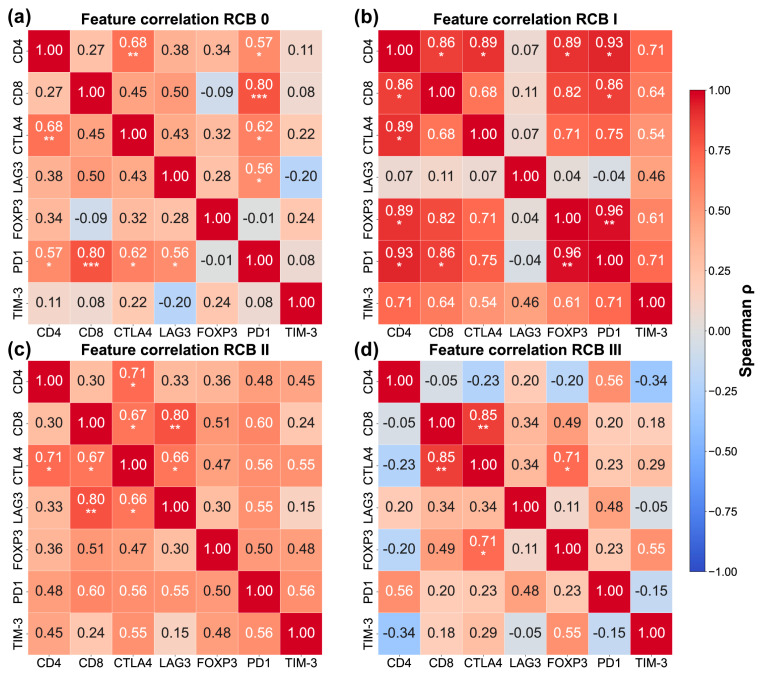
Correlation analysis of immune cell markers and immune checkpoint expression across RCB categories. Spearman correlation matrices depict relationships between immune cell markers (CD4, CD8, FOXP3) and immune checkpoint molecules (CTLA-4, LAG-3, PD1, TIM-3), stratified by pathological response to NACT: (**a**) RCB 0 (*n* = 20), (**b**) RCB I (*n* = 7), (**c**) RCB II (*n* = 15), and (**d**) RCB III (*n* = 15). Correlation coefficients (ρ) are displayed in each cell, with color intensity representing correlation strength (red = positive, blue = negative) Statistical significance is denoted by asterisks based on Benjamini–Hochberg-adjusted *p*-value (* *p* < 0.05, ** *p* < 0.01, *** *p* < 0.001). Full statistical details including uncorrected and adjusted *p*-values as well as bootstrap 95% confidence intervals (1000 resamples) are provided in Supplementary Tables S2–S5. The distinct correlation patterns across RCB categories highlight the dynamic immune coordination associated with treatment response and residual disease burden, with a progressive decrease in significant correlations from chemosensitive (RCB 0–I) to chemoresistant (RCB II–III) tumors.

Given the wide dynamic range observed across immune cell markers and immune checkpoint expression among molecular phenotypes and RCB categories, we performed a Spearman correlation analysis stratified by pathological response group (RCB 0–III; Figure 5; Supplementary Tables S2–S5). To account for multiple testing, Benjamini–Hochberg correction was applied to the pairwise correlations within each RCB stratum. Bootstrap confidence intervals (1000 resamples) quantified the stability of correlation estimates across RCB strata and are provided in Supplementary Tables S2–S5. In tumors that achieved pCR (RCB 0 *n* = 20; Figure 5a), moderate-to-strong positive correlations were observed between PD1 expression and multiple immune cell markers, including CD4 (ρ = 0.57, 95% CI [0.128, 0.885], *p* = 0.0086, BH adj. *p* = 0.0403), CD8 (ρ = 0.80, 95% CI [0.534, 0.901], *p* < 0.0001, BH adj. *p* = 0.0006), and CTLA-4 (ρ = 0.62, 95% CI [0.169, 0.899], *p* = 0.0034, BH adj. *p* = 0.0240). Notably, PD1 showed the strongest association with CD8, suggesting that PD1 expression in this group is closely linked to cytotoxic T cell infiltration. CTLA-4 also correlated positively with CD4 (ρ = 0.68, 95% CI [0.283, 0.872], *p* = 0.0009, BH adj. *p* = 0.0093) and nominally with CD8 (ρ = 0.45, 95% CI [−0.015, 0.799], *p* = 0.0441, BH adj. *p* = 0.1323, not significant after correction), while LAG3 showed a significant correlation with PD1 (ρ = 0.56, 95% CI [0.152, 0.862], *p* = 0.0096, BH adj. *p* = 0.0403). Five correlations survived false discovery rate correction (Supplementary Table S2), supporting the presence of a coordinated immune network in tumors with complete pathological response.

In RCB I (*n* = 7) tumors, a highly coordinated immune network emerged (Figure 5b). Strong positive correlations were observed among CD4, CD8, CTLA-4, FOXP3, PD1, and TIM-3. Notably, the associations between PD1 and FOXP3 (ρ = 0.96, *p* = 0.0005, 95% CI [0.698, 1.0], BH adj. *p* = 0.0095) and between CD4 and PD1 (ρ = 0.93, 95% CI [0.412, 1.0], *p* = 0.0025, BH adj. *p* = 0.0265) were particularly strong. Additional significant correlations included CD4-FOXP3 (ρ = 0.89, 95% CI [0.4, 1.0], *p* = 0.0068, BH adj. *p* = 0.0357), CD4-CTLA4 (ρ = 0.89, 95% CI [0.412, 1.0], *p* = 0.0068, BH adj. *p* = 0.0357), CD4-CD8 (ρ = 0.86, 95% CI [0.4, 1.0], *p* = 0.0137, BH adj. *p* = 0.0479), and CD8-PD1 (ρ = 0.86, 95% CI [0.333, 1.0], *p* = 0.0137, BH adj. *p* = 0.0479). Six correlations survived false discovery rate correction (Supplementary Table S3), representing the highest proportion of significant associations across all RCB groups. However, correlation estimates from small samples are inherently unstable, and these patterns require validation in larger cohorts with minimal residual disease.

In RCB II tumors (*n* = 15; Figure 5c), positive correlations remained evident but appeared more heterogeneous. CD8 showed strong associations with LAG-3 (ρ = 0.80, 95% CI [0.460, 0.928], *p* = 0.0003, BH adj. *p* = 0.0065) and CTLA-4 (ρ = 0.67, 95% CI [0.144, 0.916], *p* = 0.0066, BH adj. *p* = 0.035), while CD4 maintained a strong correlation with CTLA4 (ρ = 0.71, 95% CI [0.236, 0.957], *p* = 0.0031, BH adj. *p* = 0.0322). CTLA4 also correlated with LAG3 (ρ = 0.66, 95% CI [0.177, 0.935], *p* = 0.0068, BH adj. *p* = 0.0359). PD1 showed nominal correlations with several immune markers, including CD8 (ρ = 0.60, 95% CI [0.088, 0.945], *p* = 0.0189, BH adj. *p* = 0.0795, not significant after correction), CTLA-4 (ρ = 0.56, 95% CI [−0.022, 0.905], *p* = 0.0301, BH adj. *p* = 0.0818, not significant after correction), and TIM-3 (ρ = 0.56, 95% CI [0.123, 0.831], *p* = 0.0310, BH adj. *p* = 0.0818, not significant after correction). Four correlations survived false discovery rate correction (Supplementary Table S4). This pattern suggests a transition toward altered immune coordination as residual disease increases.

In contrast, tumors with extensive residual disease (RCB III *n* = 15; Figure 5d) displayed a marked disruption of immune coordination. Correlations between immune cell markers and immune checkpoints were generally weaker and, in some cases, negative. CD8 remained strongly correlated with CTLA-4 (ρ = 0.85, 95% CI [0.454, 0.978], *p* < 0.0001, BH adj. *p* = 0.0011), while CTLA4 also correlated with FOXP3 (ρ = 0.71, 95% CI [0.261, 0.953], *p* = 0.0030, BH adj. *p* = 0.0313). However, CD4 showed limited association with most checkpoints except for a nominal correlation with PD1 (ρ = 0.56, 95% CI [0.029, 0.910], *p* = 0.031, BH adj. *p* = 0.1767, not significant after correction). Notably, FOXP3 showed a nominal correlation with TIM-3 (ρ = 0.55, 95% CI [0.054, 0.859], *p* = 0.0337, BH adj. *p* = 0.1767, not significant after correction). PD1 exhibited reduced or inverse correlations with key immune cell populations, with no PD1-related correlations surviving false discovery rate correction. Only two correlations remained statistically significant after correction (Supplementary Table S5), the lowest proportion across all RCB groups, suggesting a decoupled or dysfunctional immune landscape in tumors resistant to NACT.

### 3.5. The Association of Immunological Markers Defines New Parameters That Are Related to the Pathological Response

Given the distinct correlation patterns identified across RCB categories, ranging from tightly coordinated immune networks in responders to disrupted immune networks in non-responders, we hypothesized that the functional relationships between immune cell populations and regulation markers, in addition to their individual expression levels alone, may be critical determinants of treatment response. To test this, we performed systematic feature generation of 80 candidate features from seven IHC markers (CD4, CD8, LAG3, CTLA4, FOXP3, PD1 and TIM-3). These features were designed to capture not only individual marker abundance (*n* = 7) but also their pairwise relationships through products (representing co-expression patterns, *n* = 21) and ratios (representing balance between immune populations, *n* = 42). Additionally, we generated T cell contextualized features (*n* = 10) that integrate immune regulatory marker expression with T cell abundance, calculated as (CD8 × Regulatory Marker)/CD4 and (CD4 × Regulatory Marker)/CD8 for each of five regulatory molecules (PD1, CTLA4, LAG3, TIM-3, FOXP3).

This design was based on the biological principle that the functional significance of immune regulatory molecule expression depends on the cellular context in which it occurs. The opposing roles of CD8+ cytotoxic T cell and CD4+ helper T cells in orchestrating anti-tumor immunity require that regulatory signals be interpreted relative to the balance between these subsets. Similarly, regulatory signals expressed on one T cell population may exert their suppressive effects in proportion to their abundance relative to the opposing effector population. Although IHC quantification measures aggregate bulk tissue positivity rather than reporting co-expression at single-cell level, normalizing regulatory marker expression to T cell subset ratios provides a tissue-level approximation of the functional immune balance, creating a useful and biologically informed metric supported by the differential correlation patterns observed across RCB groups.

For all subsequent analyses, pathological complete response (pCR; RCB 0, *n* = 20) was encoded as the negative class (0) and residual disease (RD; RCB I–III, *n* = 37) as the positive class (1). Each feature’s discriminative performance for distinguishing pCR from RD was evaluated using receiver operating characteristic (ROC) curve analysis, with features ranked by area under the curve (AUC) (Supplementary Figure S8). For the top 5 features, we identified an optimal cutoff value that maximized discriminative performance using Youden’s index, classified patients as above or below this cutoff, and used Fisher’s exact test with Benjamini–Hochberg to assess whether feature-high versus feature-low patients differed in pathological response, with the strength of the association quantified as odds ratios with 95% confidence intervals.

**Figure 6:**
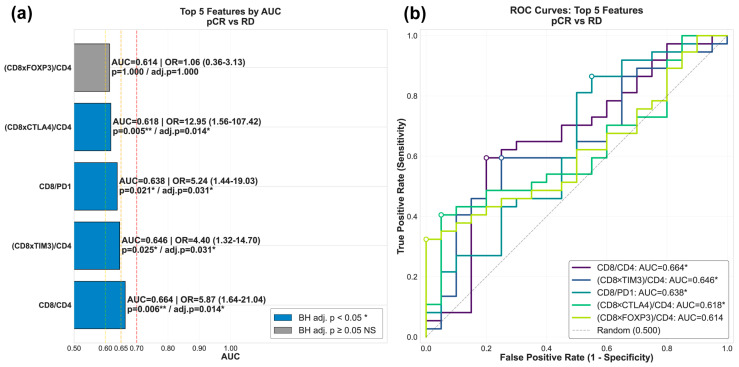
Systematic evaluation of immune features identifies predictors of NACT response. (**a**) Top 5 features ranked by area under the curve (AUC) for discriminating pathological complete response (pCR, RCB 0) from residual disease (RD: RCB I–III). Features were generated from seven IHC markers (CD4, CD8, LAG3, CTLA4, FOXP3, PD1, TIM-3) across four categories: single markers (*n* = 7), pairwise products (*n* = 21), pairwise ratios (*n* = 42), and T cell-contextualized features (*n* = 10). For each feature, patients were classified as feature-high or feature-low based on an optimal cutoff (Youden’s index). Fisher’s exact test with Benjamini–Hochberg correction assessed whether these groups differed in their likelihood of pCR versus RD. Bar color indicates statistical significance after multiple testing correction (blue: *p* < 0.05 *, gray: *p* ≥ 0.05, ** *p* < 0.01). Odds ratios (ORs) with 95% confidence intervals quantify the association between feature values and outcome; this metric is displayed along with both uncorrected and adjusted *p*-values for each feature. Vertical dashed lines at AUC = 0.60 (yellow), 0.65 (orange), and 0.70 (red) indicate thresholds of minimal, moderate, and adequate clinical utility, respectively. (**b**) Receiver operating characteristic (ROC) curves for the top five features. The dashed line represents random classification (AUC = 0.500). The CD8/CD4 ratio showed the strongest performance (AUC = 0.664, OR = 5.87, *p* = 0.0060, BH adj. *p* = 0.0140) and composite features integrating CD8 with regulatory markers normalized by CD4 dominated the top performers, demonstrating that regulatory signal context are strongly associated with NACT response. Four out of five features achieved statistical significance after correction for multiple testing.

Among the top-ranked features, composite metrics reflecting the balance between cytotoxic T cell infiltration and immune regulatory signals showed the strongest discriminative capacity for treatment response (Figure 6a,b). The CD8/CD4 ratio demonstrated the highest discriminatory capacity (AUC= 0.664, OR = 5.87, 95% CI: 1.64–21.04, *p* = 0.006, BH adj. *p* = 0.014). T cell-contextualized features integrating CD8 expression with regulatory markers also showed strong associations, including (CD8 × CTLA4)/CD4 (AUC = 0.618, OR = 12.95, 95% CI: 1.56–107.42, *p* = 0.005, BH adj. *p* = 0.0140), CD8/PD1 (AUC = 0.638, OR = 5.24, 95% CI: 1.44–19.03, *p* = 0.021, BH adj. *p* = 0.031), and (CD8 × TIM3)/CD4 (AUC = 0.646, OR = 4.40, 95% CI: 1.32–14.70, *p* = 0.025, BH adj. *p* = 0.031). The feature (CD8 × FOXP3)/CD4 showed a similar AUC (0.614, OR = 1.06, 95% CI: 0.36–3.13) but did not achieve statistical significance (*p* = 0.0800, BH adj. *p* = 0.0800). Four of the five top features achieved statistical significance after correction for multiple testing.

In contrast, individual immune checkpoint markers alone exhibited limited predictive performance (AUC ≤ 0.60) and were not represented among the top five features by AUC. These findings suggest that the functional balance between effector T cells and regulatory signals may provide added discriminative capacity for predicting treatment response to single-marker expression in this exploratory cohort.

Building on the finding that composite immune features outperformed individual markers, we next integrated the five top-ranked features into a multivariate Random Forest model to assess their combined predictive capacity for pCR (*n* = 20) vs. RD (*n* = 37). Random Forest was selected for its ability to capture non-linear relationships and interactions among features, which is an important consideration given the complex immune coordination patterns observed across RCB groups in our correlation analysis. The model incorporated CD8/CD4, (CD8 × TIM-3)/CD4, CD8/PD1, (CD8 × CTLA4)/CD4, and (CD8 × FOXP3)/CD4, along with tumor phenotype encoded as dummy variables

**Figure 7:**
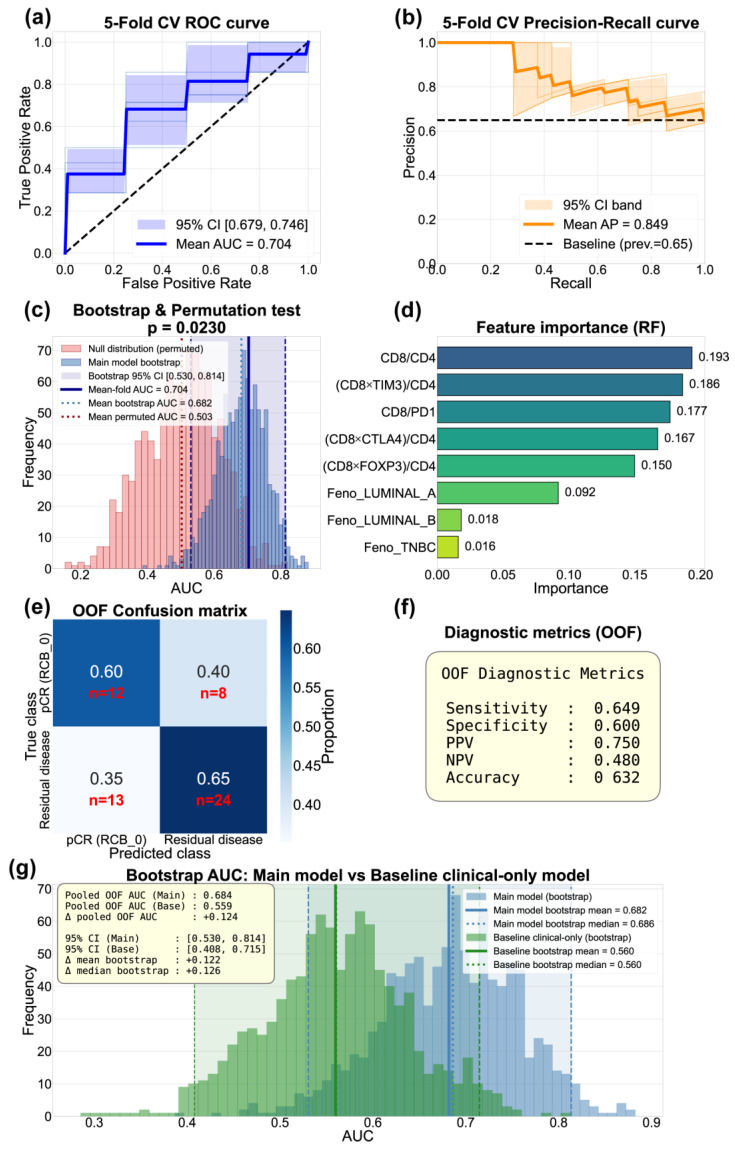
Random Forest model integrating composite immune features predicts response to NACT. (**a**) 5-fold stratified cross-validation ROC curves. Individual fold curves (light blue) and the mean ROC curve (blue line) with 95% confidence interval (blue shaded area) show the model’s performance in discriminating pCR from RD, with a cross-validated AUC = 0.704 (95% CI: 0.679–0.746). The dashed diagonal line represents random classification. (**b**) 5-Fold cross-validation precision–recall curves. Individual fold curves (light orange) and the mean precision–recall curve (orange line) with 95% confidence band (orange shaded area) demonstrate mean average precision (AP) = 0.849, substantially exceeding the baseline prevalence of 0.65 (black dashed horizontal line). (**c**) Bootstrap and permutation test results (*n* = 1000 iterations each). Pink histogram shows the null distribution of AUC values from random permutations of class labels, demonstrating expected performance under no association (mean permuted AUC = 0.503). Blue histogram shows bootstrap distribution from 1000 resamples of pooled predictions, with bootstrap mean AUC = 0.682 (95% CI: 0.530–0.814). Blue dashed vertical lines mark the bootstrap 95% CI bounds. Vertical reference lines indicate; cross-validated mean fold AUC (solid blue, 0.704), bootstrap mean AUC (blue dotted, 0.682), and mean permuted AUC (red dotted, 0.503). The observed performance significantly exceeds chance (one-sided permutation *p* = 0.0230). (**d**) Feature importance ranked by mean decrease in Gini impurity from the final model trained on the complete dataset. The top predictors are immune-derived composite features integrating CD8+ T cell abundance with regulatory markers normalized by CD4+ context, while tumor phenotype variables contribute comparatively less. (**e**) Out-of-fold (OOF) confusion matrix showing classification results aggregated across all cross-validation folds. Values represent the proportion of correctly and incorrectly classified cases, with absolute counts displayed in red. The model correctly classified 60% of pCR cases (12/20) and 65% of RD cases (24/37). (**f**) Diagnostic metrics calculated from OOF predictions, including sensitivity (64.9%), specificity (60.0%), PPV (75.0%), NPV (48.0%), and accuracy (63.2%). (**g**) Bootstrap AUC comparison of our immune-augmented main model vs. a clinical-only baseline model (*n* = 1000 bootstrap resamples each of pooled out-of-fold predictions). Blue histogram shows the immune-augmented model bootstrap distribution (bootstrap mean AUC = 0.682, bootstrap median AUC = 0.686, 95% CI: 0.530–0.814); green histogram shows the clinical-only baseline model bootstrap distribution (bootstrap mean AUC = 0.560, bootstrap median = 0.560, 95% CI: 0.408–0.715). Shaded regions and dashed vertical lines mark the 95% CI bounds for each model. Solid vertical lines indicate bootstrap means; dotted vertical lines indicate bootstrap medians. The text box displays pooled out-of-fold AUCs (Main: 0.684, Baseline: 0.559), the difference in pooled OOF AUCs (Δ pooled OOF AUC = +0.124), bootstrap 95% CIs, and differences in bootstrap means (Δ mean bootstrap = +0.122) and bootstrap medians (Δ median bootstrap = +0.126), demonstrating consistent improvement of the immune-augmented model over the clinical-only baseline in this exploratory cohort.

Using a five-fold stratified cross-validation framework, the model achieved a mean fold AUC of 0.704 (95% CI: 0.679–0.746) for discriminating pCR or from RD (Figure 7a). The precision–recall curve demonstrated a mean average precision of 0.849 (Figure 7b), substantially exceeding the baseline prevalence of 0.65, indicating robust performance in the imbalanced dataset. Cross-validated performance metrics from out-of-fold (OOF) predictions included an overall accuracy of 63.2%, sensitivity of 64.9%, specificity of 60.0%, a PPV of 75.0%, and NPV of 48.0% (Figure 7f). The OOF predictions were integrated into a confusion matrix (Figure 7e), revealing that the model correctly classified 60% of pCR cases and 65% of RD cases, demonstrating balanced performance across both outcome groups.

Monte Carlo permutation testing with 1000 iterations confirmed that the observed AUC significantly exceeded random expectation (*p* = 0.023; Figure 7c), demonstrating that the integrated immune feature signature provides meaningful predictive information for treatment response. Bootstrap resampling of out-of-fold predictions (1000 iterations) yielded a mean AUC of 0.682 and median AUC of 0.686 (95% CI: 0.530–0.814), confirming model stability across resampled datasets (Figure 7c).

Analysis of feature importance (FI) highlighted that immune-derived composite variables dominated the predictive landscape (Figure 7d). Specifically, CD8/CD4 showed the highest importance (FI = 0.193), followed by CD8 × TIM-3/CD4 (FI = 0.186), CD8/PD1 (FI = 0.177), CD8 × CTLA-4/CD4 (FI = 0.167), and CD8 × FOXP3/CD4 (FI = 0.150). While tumor phenotype variables contributed comparatively less to model performance, all top-ranking features integrated cytotoxic T cell abundance with regulatory signaling normalized by CD4+ T cell context. This reinforces the concept that the balance between effector immunity and immune regulation or exhaustion can work as a predictor of response to NACT.

To quantify the added value of immune features beyond standard clinical variables, a baseline model was trained using phenotype dummy variables alone under identical cross-validation conditions. The baseline clinical-only model achieved a pooled out-of-fold AUC of 0.559 (bootstrap mean AUC = 0.560, 95% CI: 0.408–0.715). Bootstrap comparison of the two models demonstrated consistent improvement with our immune-augmented approach (pooled ΔAUC = +0.124; bootstrap mean ΔAUC = +0.122, median ΔAUC = +0.126) (Figure 7g).

## 4. Discussion

In this exploratory study, transcriptomic immunological deconvolution approaches using RNAseq data from a previous study (GSE162187) [35] were used in conjunction with immunohistochemistry (IHC) (from an independent cohort of patients) to identify the immunological determinants of response to neoadjuvant chemotherapy (NACT) in breast cancer. Our findings suggest that the functional organization and coordination of the tumor immune microenvironment may provide added value complementary to baseline immune cell abundance and individual immune checkpoint marker expression, for understanding chemosensitivity in breast cancer. Numerous studies on cancer, particularly on breast cancer, have shown that tumors that achieve a pCR display an active immune signature; that is, if the tumor microenvironment is biased toward a cytotoxic profile (NK and T cells), the effectiveness of therapy is significantly increased [36,37]. In the study of Ignatiadis et al., they found that STAT1-related immune modules and T cell lineage are robust predictors of sensitivity to anthracycline- and taxane-based chemotherapy [38]. These findings suggest that type 1 immunity—mediated by interferon-gamma production and the activation of T and NK cells—functions as the determining mechanism for treatment-induced tumor clearance. Consistent with this framework, our deconvolution of bulk RNAseq data revealed trends toward the enrichment of NK cells, Th1-polarized CD4+ T cells, and non-regulatory CD4+ T cells in chemosensitive tumors, alongside relative macrophage depletion. Although these signals did not achieve statistical significance after multiple testing correction—likely due to the limited cohort size (*n* = 44)—the signature aligns with prior evidence linking innate and adaptive immune activation to chemotherapy efficacy [39]. The relevance of this immune activation has also been stablished in the context of residual disease following NACT, particularly in TNBC. For instance, Pérez-Pena et al. utilized transcriptomic profiling to demonstrate that immune activation in residual disease is associated with a better response to adjuvant chemotherapy [40]. Similarly, Blaye et al. reported that a deficient immune response, primarily involving lymphocytes, correlates with distant tumor progression in patients with residual disease [41]. Beyond immunological factors, tumor burden remains critical: residual TNBC tumors larger than 18 mm have been associated with poorer survival [42]. Collectively, these findings underscore the prognostic and predictive value of using transcriptomic analysis to characterize the immune landscape and its impact on tumor response.

Despite extensive research into genetic and transcriptomic markers for chemotherapy response in breast cancer, few diagnostic tests have reached clinical application [43,44]. Many existing assays require specialized procedures that are difficult to implement in resource-limited healthcare settings [45,46]. However, the growing evidence linking TILs to pCR justifies further investigation into accessible implementation strategies, particularly those leveraging existing infrastructure like IHC services [47]. The IHC results of this work showed that across molecular phenotypes, pathological response followed expected patterns: Luminal B and TNBC tumors more frequently achieved pCR, while Luminal A tumors showing higher residual disease burden [48]. However, immune infiltration, as assessed by CD4, CD8 and FOXP3 expression, did not differ significantly between phenotypes or RCB classes. Similarly, expression levels of individual immune checkpoint markers (LAG3, PD1, CTLA4, TIM-3) were not significantly different across response groups after Benjamini–Hochberg correction. Together, these observations suggest that while molecular phenotype remains a strong determinant of pathological response to chemotherapy, baseline immune infiltration as measured by conventional IHC does not substantially differ across phenotypes in this cohort, indicating that immune cell abundance alone may not fully explain differential treatment response and underscoring the potential value of integrating additional immune contexture metrics. A key observation was that markers like PD1 and CTLA-4, displayed a wide dynamic range of expression across tumors, independent of phenotype or RCB category. This heterogeneity suggests that immune functional states vary substantially within clinically defined subgroups, potentially explaining the variable treatment responses observed even among tumors with similar molecular classifications [49]. These findings support the concept that functional immune heterogeneity can exist even in the absence of marked quantitative differences in T cell infiltration, suggesting that immune contexture is defined not only by cell abundance but also by functional states reflected in immune regulatory marker expression. Our findings differ from prior studies that demonstrated significant associations between immune cell abundance and treatment response. Miyashita et al. demonstrate that high CD8(+) TIL and a high CD8+/FOXP3+ ratio in residual tumors accurately predict pCR in TNBC patients following NAC [50]. In addition, Zhang et al. showed that FOXP3+/CD25+ ratio was positively correlated with better overall survival in TNBC [51]. These differences likely reflect methodological variation in our approaches, differences in patient populations and sample sizes, or the specific timing of immune assessment. Notably, despite these methodological differences, both our findings and these prior studies converge on a consistent principle; relative immune balance, captured through ratios and contextualized metrics, provides valuable information for understanding treatment response.

Overall, PD1, CTLA-4, and TIM-3 are not expressed in a strict sequential order, instead, they appear at different stages and contexts, often co-expressed on exhausted T cells. PD1 typically emerges early during T cell activation, CTLA-4 regulates initial immune responses, and TIM-3 often co-expresses with PD1 as T cells become severely exhausted, with this combination signaling profound functional impairment in the face of chronic antigen exposure [52,53]. Thus, these molecules reflect a spectrum of T cell activation and exhaustion states.

A key insight from our study emerges from the Spearman correlation analyses of immune cell markers and immune checkpoint expression stratified by pathological response (RCB 0–III). By focusing on the patterns of immune interactions in addition to absolute expression, this analysis was conducted within each response category, revealing distinct coordination patterns in immune organization that were not evident from single-marker comparations. Importantly, all tumor samples analyzed in this study were obtained from pre-treatment biopsies, meaning that these correlation patterns reflect baseline immune states potentially predictive of subsequent chemotherapy response, not a treatment-induced effect. Across RCB groups, the proportion of correlations surviving false discovery rate correction decreased progressively from chemosensitive to chemoresistant tumors (RCB 0: 5/21, RCB I: 6/21, RCB II: 4/21, RCB III: 2/21), with a notable shift from PD1-centered networks in responders to reduced immune coordination in non-responders. These correlation analyses suggest that treatment response is associated not merely with the magnitude of immune infiltration or checkpoint expression, but with the underlying coordination of immune interactions at baseline. This highlights the potential value of evaluating immune checkpoints in the context of immune cell composition to better capture immune states linked to chemotherapy sensitivity or resistance.

In tumors that subsequently achieved a pCR (RCB 0, *n* = 20), we observed strong positive correlations between PD1 and CD8 (ρ = 0.80, *p* < 0.0001, BH adj. *p* = 0.0006). This suggests baseline PD1 expression in these tumors may signify pre-existing activated cytotoxic T cell infiltration in addition to checkpoint-mediated regulation. An even more pronounced pattern was observed in tumors with minimal residual disease (RCB I, *n* = 7), which exhibited the highest degree of baseline immune coordination across all groups. Strong correlations were found among CD4, CD8, FOXP3, PD1, CTLA-4, and TIM-3, with particularly high associations between PD1 and FOXP3 (ρ = 0.96, *p* = 0.0005, BH adj. *p* = 0.0095) and between CD4 and PD1 (ρ = 0.93, *p* = 0.0025, BH adj. *p* = 0.0265). Concurrent positive correlations between CTLA-4 and both CD4 (ρ = 0.89, *p* = 0.0068, BH adj. *p* = 0.0357) and CD8 (ρ = 0.68, *p* = 0.094, BH adj. *p* = 0.164) further suggest that tumor with favorable response harbor pre-treatment immune microenvironments characterized by coordinated T cell activation and regulation, which may primer the tumor for effective response to chemotherapy. However, given the very small sample size in RCB I, these correlation estimates are inherently unstable and require validation in larger cohorts with minimal residual disease. Bootstrap confidence interval analysis (1000 resamples) quantified this instability, with several RCB I correlations showing very wide confidence intervals (e.g., CD4-PD1: 95% CI: 0.412–1.0; CD8-PD1: 95% CI: 0.333–1.0), confirming substantial uncertainty in these estimates despite high point correlations.

Recent advances in T cell biology have demonstrated that the classical view of T cell exhaustion as a terminal state has evolved with the identification of multiple intermediate exhaustion phenotypes, particularly within CD8+ T cell populations [54,55,56]. These subsets, including progenitor exhausted cells, retain proliferative capacity and are important for sustained anti-tumor responses [57]. TIM-3 and PD1 are classically described as markers of T cell exhaustion in settings of chronic antigen stimulation, such as persistent viral infection and cancer. In these contexts, sustained TIM-3 expression, particularly when co-expressed with PD1, has been linked to impaired cytokine production, reduced proliferative capacity, and defective T cell signaling, correlating with high antigen load and disease progression [58]. However, accumulating evidence indicates that TIM-3 and PD1 expressions are context-dependent and do not uniformly signify terminal dysfunction.

During acute or highly immunogenic stimulation, TIM-3 can be transiently expressed on activated effector CD8+ T cells [59,60]. Several studies have described CD8+ T cells with a “restrained” or “transitory” phenotype that simultaneously express exhaustion-associated markers, such as PD1 and TIM-3 while retaining key effector functions, including granzyme B production, cytotoxic capacity, and expression of transcription factors associated with effector differentiation [61,62]. These populations have been identified in cancer, chronic infection, and autoimmunity, suggesting that checkpoint expression does not necessarily indicate functional impairment but can mark cells with preserved anti-tumor potential [63].

These emerging concepts of intermediate exhaustion immunophenotypes align with our observations of pre-treatment immune organization. In tumors that subsequently achieved pCR (RCB 0, *n* = 20) or minimal residual disease (RCB I, *n* = 7), immune checkpoints such as PD1, CTLA-4, and TIM-3 showed positive correlations with CD8 and CD4 T cell markers, suggesting that pre-treatment checkpoint expression may reflect ongoing immune activation and regulation alongside checkpoint-mediated control, contributing to a functional immune microenvironment.

Importantly, the pre-treatment tumor microenvironment in breast cancer represents a setting of evolving immune surveillance prior to the rapid tumor cell death and antigen release induced by chemotherapy. In this context, the coordinated baseline expression patterns we observed, particularly TIM-3 and PD1 expression on CD8+ T cells occurring alongside robust T cell infiltration, may indicate the presence of activated T cell populations prepared for effective anti-tumor response upon chemotherapeutic challenge, potentially representing functional immune states capable of responding to treatment-induced immunogenic signals, this interpretation is consistent with the concept that checkpoint expression in the pre-treatment setting may reflect immune engagement with tumor antigens in addition to regulatory mechanisms [39].

The wide dynamic range of checkpoint expressions observed across RCB categories, particularly for PD1 and CTLA-4, further supports the presence of heterogeneous CD8+ T cell states within the tumor microenvironment, potentially encompassing intermediate exhaustion phenotypes that retain effector function. Although our IHC data measures aggregate tissue positivity rather than single cell co-expression, the performance of composite features, particularly those integrating CD8 expression levels with checkpoint markers normalized by CD4 context, suggests that these tissue-level metrics may capture functionally meaningful immune states that provide added value beyond single-marker analyses.

Together, these findings support a model in which baseline immune microenvironments characterized by coordinated CD8+ T cell states with concurrent effector and regulatory features are associated with NACT response, whereas disrupted immune coordination at baseline may predict resistance. This framework provides a biological explanation for why immune balance and coordination at baseline, beyond immune infiltration or checkpoint expression alone, emerge as key determinants of pathological response in our study.

Extensive evidence, including multiple meta-analyses, supports a positive association between higher stromal tumor-infiltrating lymphocytes (TILs), increased CD8+ T cell density, and improved pathological complete response rates, particularly in triple-negative and HER2-positive breast cancer [22,23]. Our findings do not contradict this established literature. Rather, the absence of statistically significant differences in immune cell abundance across response groups in our cohort may reflect limited sample size, methodological variation in IHC quantification, or differences in the patient populations studied. Our results suggest that, within this context, immune coordination and functional organization may provide additional, complementary information beyond immune abundance alone, helping to explain variability in chemotherapy response among tumor with comparable levels of immune infiltration.

Building on the observed importance of immune coordination, we found that integrating T cell infiltration metrics with immune regulatory marker expression into composite features improved the ability to predict pathological response to NACT in this cohort. The Random Forest model incorporating the top five features along with tumor phenotype achieved cross-validated mean AUC of 0.704 (95% CI: 0.679–0.746), with a pooled out-of-fold AUC of 0.684. To assess model stability and quantify uncertainty, we performed bootstrap resampling (1000 iterations) of out-of-fold predictions, yielding a bootstrap mean AUC of 0.682 (95% CI: 0.530–0.814), confirming consistent performance across resampled datasets. Monte Carlo permutation testing (1000 iterations) demonstrated that this performance significantly exceeded chance expectation (*p* = 0.023).

To quantify the added value of immune features beyond standard clinical variables, we trained a baseline model using molecular phenotype alone under identical cross-validation conditions. The baseline clinical-only model achieved a pooled out-of-fold AUC of 0.559 (bootstrap mean AUC = 0.560, 95% CI: 0.408–0.715). Bootstrap comparison of the two models demonstrated consistent improvement with the immune-augmented approach across 1000 resamples (pooled ΔAUC = +0.124; bootstrap mean ΔAUC = +0.122, median ΔAUC = +0.126). The consistent separation observed across bootstrap iterations provides evidence that immune features contribute meaningful discriminative information beyond molecular phenotype in this exploratory setting.

Feature importance analysis revealed that immune-derived composite variables dominated the predictive signal, with CD8/CD4 showing the highest importance (0.193), followed by T cell-contextualized features integrating checkpoint expression with T cell balance. In contrast, molecular phenotype variables contributed comparatively less (Luminal A: 0.092, Luminal B: 0.018, TNBC: 0.016), indicating that immune coordination metrics provide substantial added value for understanding treatment response variability. This model is an exploratory proof-of-concept, where we found that pre-treatment immune contexture captures predictive information, and warrants validation in larger, independent cohorts as a potential component of clinical decision support tools.

Limitations of our study include our relatively small (*n* = 57), single-center retrospective cohort, which limits statistical power and generalizability, especially for subtypes like HER2+ (*n* = 6). Stratification into four RCB categories further reduces statistical power within each group, particularly for RCB I (*n* = 7). Bootstrap confidence intervals confirmed this limitation, revealing wide uncertainty ranges for correlation estimates in small strata. Accordingly, the absence of statistically significant differences across some RCB strata should be interpreted with caution, as it may reflect limited sample size rather than true biological equivalence.

Regarding our modeling approach, feature selection was performed on the full dataset, prior to Random Forest training and cross-validation. To assess potential optimism from this strategy, we applied bootstrap resampling (1000 iterations) of out-of-fold predictions, which yielded a bootstrap mean AUC of 0.682 compared to the cross-validated mean fold AUC of 0.704, suggesting modest optimism of 0.02. Such modest optimism likely reflects with our hypothesis-driven feature engineering based on immunological principles with a focused feature space, rather than derived from exhaustive combinatorial search. The biological convergence of multiple validation approaches permutation testing, bootstrap stability and baseline comparison are consistent with a genuine, yet modest, predictive signal in this exploratory cohort. This Random Forest model, while achieving statistically significant discriminative performance in this exploratory framework, requires validation in larger, prospective, and multicenter cohorts to confirm generalizability. The composite features generated represent tissue-level approximations of functional immune balance, our IHC cannot confirm single-cell co-expression of the markers. Therefore, future studies employing multiplex IHC or technologies aimed at determining molecular co-expression, are needed to validate the existence of cells co-expressing these marker combinations within the tumor microenvironment before treatment. Despite these constraints, it is important to emphasize the practical relevance of our findings. The decision to use standard IHC was guided by the necessity to facilitate the translation of our observations and analytical methods into routine diagnostic practice. The immune features identified in this study can be assessed using existing clinical infrastructure and provide a framework for investigating immune coordination as a predictor of chemotherapy response in breast cancer patients.

In conclusion, our findings represent a step forward in understanding the immunological determinants of chemotherapy response in breast cancer. Immune balance features such as CD8/CD4 ratio and T cell-contextualized metrics dominated the model’s feature importance, suggesting that pre-treatment immune coordination provides valuable information complementary to current clinical classifiers in this setting. While our Random Forest model demonstrated added discriminative value over molecular phenotype alone (bootstrap mean ΔAUC = +0.122), the modest sample size, and exploratory framework calls for a validation in larger, independent cohorts before clinical implementation.

Future work should focus on this validation and integrating pre-treatment immune contexture with clinical and molecular data to refine patient stratification and guide the development of treatment strategies pairing chemotherapy with immunomodulatory agents designed for specific immune states. Our planned next steps include applying this model to larger patient cohorts to evaluate its performance in expanded clinical settings with the long-term goal of improving treatment selection and outcomes for patients with locally advanced breast cancer receiving neoadjuvant chemotherapy.

## Data Availability

The original contributions presented in this study are included in the article. Further inquiries can be directed to the corresponding authors.

## Supplementary Materials

The following supporting information can be downloaded at https://www.mdpi.com/article/10.3390/biomedicines14030663/s1. Table S1: Clinical and pathological characteristics of the study cohort (*n* = 57); Table S2: Spearman correlation analysis of immune markers in tumors achieving pathological complete response (pCR, RCB 0); Table S3: Spearman correlation analysis of immune markers in tumors with minimal residual disease (RCB I); Table S4: Spearman correlation analysis of immune markers in tumors with moderate residual disease (RCB II); Table S5: Spearman correlation analysis of immune markers in tumors with extensive residual disease (RCB III); Supplementary Figure S1: Representative images of CD4+ cell detection in tumor biopsies from breast cancer patients; Supplementary Figure S2: Representative images of CD8+ cell detection in tumor biopsies from breast cancer patients; Supplementary Figure S3: Representative images of FoxP3-positive cell detection in tumor biopsies from breast cancer patients; Supplementary Figure S4: Representative images of LAG3-positive cell detection in tumor biopsies from breast cancer patients; Supplementary Figure S5: Representative images of PD1-positive cell detection in tumor biopsies from breast cancer patients; Supplementary Figure S6: Representative images of CTLA4-positive cell detection in tumor biopsies from breast cancer patients; Supplementary Figure S7: Representative images of TIM-3-positive cell detection in tumor biopsies from breast cancer patients; Supplementary Figure S8: Immune features ranked by AUC.
