# Supplementary material for: A Predictive Immunological Signature Associated with Pathological Response in Breast Cancer Treated with Neoadjuvant Chemotherapy
Source: Biomedicines. 2026 Mar 14;14(3):663. doi: 10.3390/biomedicines14030663 (PMC13023440; doi:10.3390/biomedicines14030663)
Supplement: Supplementary file 1 [file biomedicines-14-00663-s001.zip › Table S1.pdf]

Table S1. Clinical and pathological characteristics of the study cohort.

Patient	Age	Molecular subtype	Stage	TNM	RCB	Status
BC01	42	LUMINAL_A	IIIA	T3N1M1	RCB_0	pCR
BC02	79	LUMINAL_A	IIIA	T1N2MX	RCB_I	Residual disease
BC03	65	LUMINAL_A	IIIA	T1N2M0	RCB_II	Residual disease
BC04	63	LUMINAL_A	IIIB	T4N2MX	RCB_II	Residual disease
BC05	34	LUMINAL_A	IIIB	T4N2MX	RCB_II	Residual disease
BC06	64	LUMINAL_A	IIIA	T3N1MX	RCB_II	Residual disease
BC07	46	LUMINAL_A	IIIA	T3N1MX	RCB_III	Residual disease
BC08	87	LUMINAL_A	IIIB	T4N2MX	RCB_III	Residual disease
BC09	57	LUMINAL_A	IIIA	T2N2MX	RCB_III	Residual disease
BC10	64	LUMINAL_A	IIIA	T3N1MX	RCB_III	Residual disease
BC11	38	LUMINAL_A	IIIA	T3N1MX	RCB_III	Residual disease
BC12	46	LUMINAL_A	IIIB	T3N2M0	RCB_III	Residual disease
BC13	52	LUMINAL_A	IIIB	T4N0M0	RCB_III	Residual disease
BC14	33	LUMINAL_B	IIA	T2N0MX	RCB_0	pCR
BC15	44	LUMINAL_B	IIIC	T4N3MX	RCB_0	pCR
BC16	61	LUMINAL_B	IIB	T2N1M0	RCB_0	pCR
BC17	59	LUMINAL_B	IIIA	T3N1M0	RCB_0	pCR
BC18	47	LUMINAL_B	IIIC	T3N3M0	RCB_0	pCR
BC19	65	LUMINAL_B	IIIB	T4N1MX	RCB_0	pCR
BC20	29	LUMINAL_B	IIA	T2N0MX	RCB_0	pCR
BC21	26	LUMINAL_B	IIIA	T3N1MX	RCB_I	Residual disease
BC22	45	LUMINAL_B	IIA	T2N0MX	RCB_I	Residual disease
BC23	55	LUMINAL_B	IIIB	T2N1M0	RCB_I	Residual disease
BC24	66	LUMINAL_B	IIIA	T3N1MX	RCB_II	Residual disease
BC25	59	LUMINAL_B	IIIC	T4N3MX	RCB_II	Residual disease
BC26	31	LUMINAL_B	IIB	T1M1M0	RCB_II	Residual disease
BC27	43	LUMINAL_B	IIB	T3N0M0	RCB_II	Residual disease
BC28	44	LUMINAL_B	IIIA	T3N2M0	RCB_II	Residual disease

BC29	41	LUMINAL_B	IIIA	T3N1M0	RCB_III	Residual disease
BC30	43	LUMINAL_B	IIIC	T3N3M0	RCB_III	Residual disease
BC31	59	LUMINAL_B	IIIA	T3N2MX	RCB_III	Residual disease
BC32	46	LUMINAL_B	IIIA	T2N2M0	RCB_III	Residual disease
BC33	47	HER2	IIIA	T3N1M0	RCB_0	pCR
BC34	60	HER2	IIIA	T2N2MX	RCB_0	pCR
BC35	60	HER2	IIIA	T3N1M0	RCB_0	pCR
BC36	51	HER2	IIIA	T3N1MX	RCB_0	pCR
BC37	48	HER2	IIB	T2N1MX	RCB_0	pCR
BC38	49	HER2	lllA	T3N2M0	RCB_III	Residual disease
BC39	57	TNBC	IIB	T2N1MX	RCB_0	pCR
BC40	46	TNBC	IIA	T2N0MX	RCB_0	pCR
BC41	55	TNBC	IIB	T2N1M0	RCB_0	pCR
BC42	37	TNBC	IIIA	T2N1M0	RCB_0	pCR
BC43	48	TNBC	IIIA	T3N1MX	RCB_0	pCR
BC44	37	TNBC	IIIA	T3N2M1	RCB_0	pCR
BC45	53	TNBC	IIB	T2N1MX	RCB_0	pCR
BC46	43	TNBC	IIIA	T2N2MX	RCB_I	Residual disease
BC47	70	TNBC	IIIB	T4N1MX	RCB_I	Residual disease
BC48	39	TNBC	IIIA	T3N1MX	RCB_I	Residual disease
BC49	46	TNBC	IIIB	T2N2MX	RCB_II	Residual disease
BC50	37	TNBC	IIA	T3N1MX	RCB_II	Residual disease
BC51	34	TNBC	IIIB	T3N2MX	RCB_II	Residual disease
BC52	36	TNBC	IIIB	T4N2MX	RCB_II	Residual disease
BC53	58	TNBC	IIIA	T2N2MX	RCB_II	Residual disease
BC54	60	TNBC	IIIB	T3N2MX	RCB_II	Residual disease
BC55	43	TNBC	IIIA	T3N1M0	RCB_III	Residual disease
BC56	36	TNBC	IIIC	T2N3MX	RCB_III	Residual disease
BC57	46	TNBC	IIIB	T4N2M0	RCB_III	Residual disease
