# Supplementary material for: A Predictive Immunological Signature Associated with Pathological Response in Breast Cancer Treated with Neoadjuvant Chemotherapy
Source: Biomedicines. 2026 Mar 14;14(3):663. doi: 10.3390/biomedicines14030663 (PMC13023440; doi:10.3390/biomedicines14030663)
Supplement: Supplementary file 1 [file biomedicines-14-00663-s001.zip › Table S2.pdf]

Table S2. Spearman correlation analysis of immune markers in tumors achieving pathological complete response (pCR, RCB 0).

Group	*Marker 1*	*Marker 2*	*rho*	*CI 95% lower*	*CI 95% upper*	*p*	*sig*	*p adj*	*sig adj*
RCB_0	CD4	CD8	0.2722	-0.2559	0.7229	0.2457		0.3968	
RCB_0	CD4	CTLA4	0.684	0.283	0.8717	0.0009	***	0.0093	**
RCB_0	CD4	LAG3	0.3779	-0.1248	0.7538	0.1005		0.2344	
RCB_0	CD4	FOXP3	0.3392	-0.0695	0.6273	0.1434		0.3012	
RCB_0	CD4	PD1	0.5709	0.1278	0.8846	0.0086	**	0.0403	*
RCB_0	CD4	TIM-3	0.1128	-0.3865	0.5781	0.6359		0.7852	
RCB_0	CD8	CTLA4	0.4545	-0.0147	0.7986	0.0441	*	0.1323	
RCB_0	CD8	LAG3	0.496	0.0114	0.8385	0.0261	*	0.0914	
RCB_0	CD8	FOXP3	-0.0865	-0.5411	0.4298	0.7169		0.7852	
RCB_0	CD8	PD1	0.795	0.534	0.9009	0	***	0.0006	***
RCB_0	CD8	TIM-3	0.0812	-0.393	0.5559	0.7336		0.7852	
RCB_0	CTLA4	LAG3	0.4256	-0.1511	0.7566	0.0614		0.1611	
RCB_0	CTLA4	FOXP3	0.3177	-0.0699	0.631	0.1723		0.329	
RCB_0	CTLA4	PD1	0.6218	0.1687	0.8994	0.0034	**	0.024	*
RCB_0	CTLA4	TIM-3	0.2205	-0.3135	0.6477	0.3503		0.4904	
RCB_0	LAG3	FOXP3	0.2809	-0.2518	0.6752	0.2303		0.3968	
RCB_0	LAG3	PD1	0.564	0.1523	0.8621	0.0096	**	0.0403	*
RCB_0	LAG3	TIM-3	-0.2047	-0.6482	0.272	0.3865		0.5073	
RCB_0	FOXP3	PD1	-0.0075	-0.4882	0.4205	0.9749		0.9749	
RCB_0	FOXP3	TIM-3	0.2369	-0.1946	0.603	0.3145		0.4718	
RCB_0	PD1	TIM-3	0.0767	-0.475	0.5982	0.7478		0.7852	
