# Supplementary material for: A Predictive Immunological Signature Associated with Pathological Response in Breast Cancer Treated with Neoadjuvant Chemotherapy
Source: Biomedicines. 2026 Mar 14;14(3):663. doi: 10.3390/biomedicines14030663 (PMC13023440; doi:10.3390/biomedicines14030663)
Supplement: Supplementary file 1 [file biomedicines-14-00663-s001.zip › Table S3.pdf]

Table S3. Spearman correlation analysis of immune markers in tumors with minimal residual disease (RCB I)

Group	*Marker 1*	*Marker 2*	*rho*	*CI 95% lower*	*CI 95% upper*	*p*	*sig*	*p adj*	*sig adj*
RCB_I	CD4	CD8	0.8571	0.4	1	0.0137	*	0.0479	*
RCB_I	CD4	CTLA4	0.8929	0.4118	1	0.0068	**	0.0357	*
RCB_I	CD4	LAG3	0.0714	-1	0.7504	0.879		0.9394	
RCB_I	CD4	FOXP3	0.8929	0.4	1	0.0068	**	0.0357	*
RCB_I	CD4	PD1	0.9286	0.4118	1	0.0025	**	0.0265	*
RCB_I	CD4	TIM-3	0.7143	-0.125	1	0.0713		0.1362	
RCB_I	CD8	CTLA4	0.6786	-0.059	1	0.0938		0.1641	
RCB_I	CD8	LAG3	0.1071	-0.9608	1	0.8192		0.9394	
RCB_I	CD8	FOXP3	0.8214	-0.0667	1	0.0234	*	0.0703	
RCB_I	CD8	PD1	0.8571	0.3333	1	0.0137	*	0.0479	*
RCB_I	CD8	TIM-3	0.6429	-0.2609	1	0.1194		0.1929	
RCB_I	CTLA4	LAG3	0.0714	-1	0.75	0.879		0.9394	
RCB_I	CTLA4	FOXP3	0.7143	-0.1765	1	0.0713		0.1362	
RCB_I	CTLA4	PD1	0.75	-0.0588	1	0.0522		0.1362	
RCB_I	CTLA4	TIM-3	0.5357	-0.4361	1	0.2152		0.3013	
RCB_I	LAG3	FOXP3	0.0357	-0.8824	0.8868	0.9394		0.9394	
RCB_I	LAG3	PD1	-0.0357	-0.8889	0.8667	0.9394		0.9394	
RCB_I	LAG3	TIM-3	0.4643	-0.3211	1	0.2939		0.3858	
RCB_I	FOXP3	PD1	0.9643	0.6981	1	0.0005	***	0.0095	**
RCB_I	FOXP3	TIM-3	0.6071	-0.4	1	0.1482		0.2223	
RCB_I	PD1	TIM-3	0.7143	-0.0588	1	0.0713		0.1362	
