# Supplementary material for: A Predictive Immunological Signature Associated with Pathological Response in Breast Cancer Treated with Neoadjuvant Chemotherapy
Source: Biomedicines. 2026 Mar 14;14(3):663. doi: 10.3390/biomedicines14030663 (PMC13023440; doi:10.3390/biomedicines14030663)
Supplement: Supplementary file 1 [file biomedicines-14-00663-s001.zip › Table S4.pdf]

Table S4. Spearman correlation analysis of immune markers in tumors with moderate residual disease (RCB II).

Group	*Marker 1*	*Marker 2*	*rho*	*CI 95% lower*	*CI 95% upper*	*p*	*sig*	*p adj*	*sig adj*
RCB_II	CD4	CD8	0.2985	-0.3715	0.8478	0.2799		0.3093	
RCB_II	CD4	CTLA4	0.7093	0.2363	0.9567	0.0031	**	0.0322	*
RCB_II	CD4	LAG3	0.3253	-0.2925	0.8361	0.2368		0.2925	
RCB_II	CD4	FOXP3	0.3557	-0.1484	0.6747	0.1932		0.2536	
RCB_II	CD4	PD1	0.4826	0.0324	0.7836	0.0685		0.1111	
RCB_II	CD4	TIM-3	0.4468	-0.0455	0.8001	0.095		0.1329	
RCB_II	CD8	CTLA4	0.6667	0.1435	0.9164	0.0066	**	0.0359	*
RCB_II	CD8	LAG3	0.8036	0.4597	0.9277	0.0003	***	0.0065	**
RCB_II	CD8	FOXP3	0.5107	-0.0555	0.8621	0.0517		0.1086	
RCB_II	CD8	PD1	0.5964	0.0875	0.9452	0.0189	*	0.0795	
RCB_II	CD8	TIM-3	0.2393	-0.3457	0.7332	0.3904		0.4099	
RCB_II	CTLA4	LAG3	0.6649	0.1767	0.9346	0.0068	**	0.0359	*
RCB_II	CTLA4	FOXP3	0.4701	-0.0555	0.7989	0.077		0.1156	
RCB_II	CTLA4	PD1	0.5594	-0.0218	0.9053	0.0301	*	0.0818	
RCB_II	CTLA4	TIM-3	0.5505	0.0127	0.8337	0.0335	*	0.0818	
RCB_II	LAG3	FOXP3	0.3036	-0.2758	0.8103	0.2714		0.3093	
RCB_II	LAG3	PD1	0.5464	0.0363	0.9134	0.0351	*	0.0818	
RCB_II	LAG3	TIM-3	0.15	-0.411	0.6182	0.5936		0.5936	
RCB_II	FOXP3	PD1	0.5	-0.1035	0.8545	0.0577		0.1102	
RCB_II	FOXP3	TIM-3	0.4821	-0.0501	0.7873	0.0687		0.1111	
RCB_II	PD1	TIM-3	0.5571	0.1229	0.831	0.031	*	0.0818	
