# Supplementary material for: A Predictive Immunological Signature Associated with Pathological Response in Breast Cancer Treated with Neoadjuvant Chemotherapy
Source: Biomedicines. 2026 Mar 14;14(3):663. doi: 10.3390/biomedicines14030663 (PMC13023440; doi:10.3390/biomedicines14030663)
Supplement: Supplementary file 1 [file biomedicines-14-00663-s001.zip › Table S5.pdf]

Table S5. Spearman correlation analysis of immune markers in tumors with extensive residual disease (RCB, III)

Group	*Marker 1*	*Marker 2*	*rho*	*CI 95% lower*	*CI 95% upper*	*p*	*sig*	*p adj*	*sig adj*
RCB_III	CD4	CD8	-0.0465	-0.5962	0.5269	0.8694		0.8695	
RCB_III	CD4	CTLA4	-0.2252	-0.7382	0.3479	0.4197		0.6333	
RCB_III	CD4	LAG3	0.2038	-0.3809	0.7505	0.4664		0.6333	
RCB_III	CD4	FOXP3	-0.1966	-0.6292	0.378	0.4825		0.6333	
RCB_III	CD4	PD1	0.5564	0.0289	0.9097	0.0313	*	0.1767	
RCB_III	CD4	TIM-3	-0.3414	-0.8037	0.2948	0.213		0.5162	
RCB_III	CD8	CTLA4	0.8536	0.4535	0.9783	0.0001	***	0.0011	**
RCB_III	CD8	LAG3	0.3429	-0.3408	0.8048	0.2109		0.5162	
RCB_III	CD8	FOXP3	0.4893	-0.0708	0.8473	0.0642		0.2565	
RCB_III	CD8	PD1	0.2002	-0.4428	0.76	0.4744		0.6333	
RCB_III	CD8	TIM-3	0.1821	-0.3686	0.6963	0.5159		0.6373	
RCB_III	CTLA4	LAG3	0.3357	-0.3492	0.8001	0.2212		0.5162	
RCB_III	CTLA4	FOXP3	0.7107	0.2612	0.9526	0.003	**	0.0313	*
RCB_III	CTLA4	PD1	0.2341	-0.3913	0.7701	0.401		0.6333	
RCB_III	CTLA4	TIM-3	0.2893	-0.2906	0.7433	0.2957		0.6209	
RCB_III	LAG3	FOXP3	0.1107	-0.5218	0.6776	0.6945		0.7676	
RCB_III	LAG3	PD1	0.4754	-0.1943	0.907	0.0733		0.2565	
RCB_III	LAG3	TIM-3	-0.0464	-0.5128	0.5083	0.8695		0.8695	
RCB_III	FOXP3	PD1	0.2341	-0.3602	0.696	0.401		0.6333	
RCB_III	FOXP3	TIM-3	0.55	0.0537	0.8587	0.0337	*	0.1767	
RCB_III	PD1	TIM-3	-0.1466	-0.6498	0.427	0.6022		0.7026	
