# Supplementary figures and images for: A Predictive Immunological Signature Associated with Pathological Response in Breast Cancer Treated with Neoadjuvant Chemotherapy
Source: Biomedicines. 2026 Mar 14;14(3):663. doi: 10.3390/biomedicines14030663 (PMC13023440; doi:10.3390/biomedicines14030663)

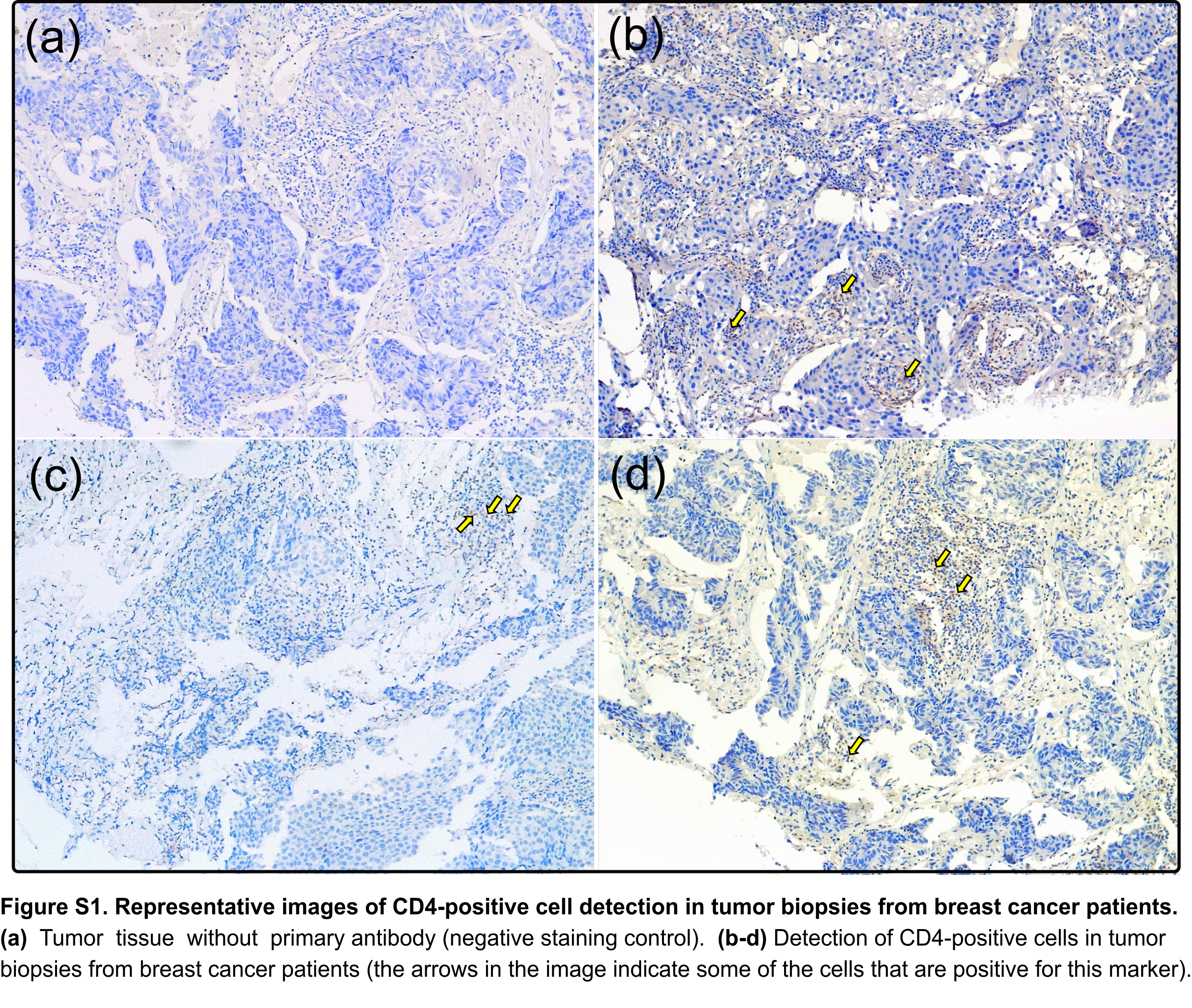

Supplement: Supplementary file 1 [file biomedicines-14-00663-s001.zip › Figure S1.tiff]

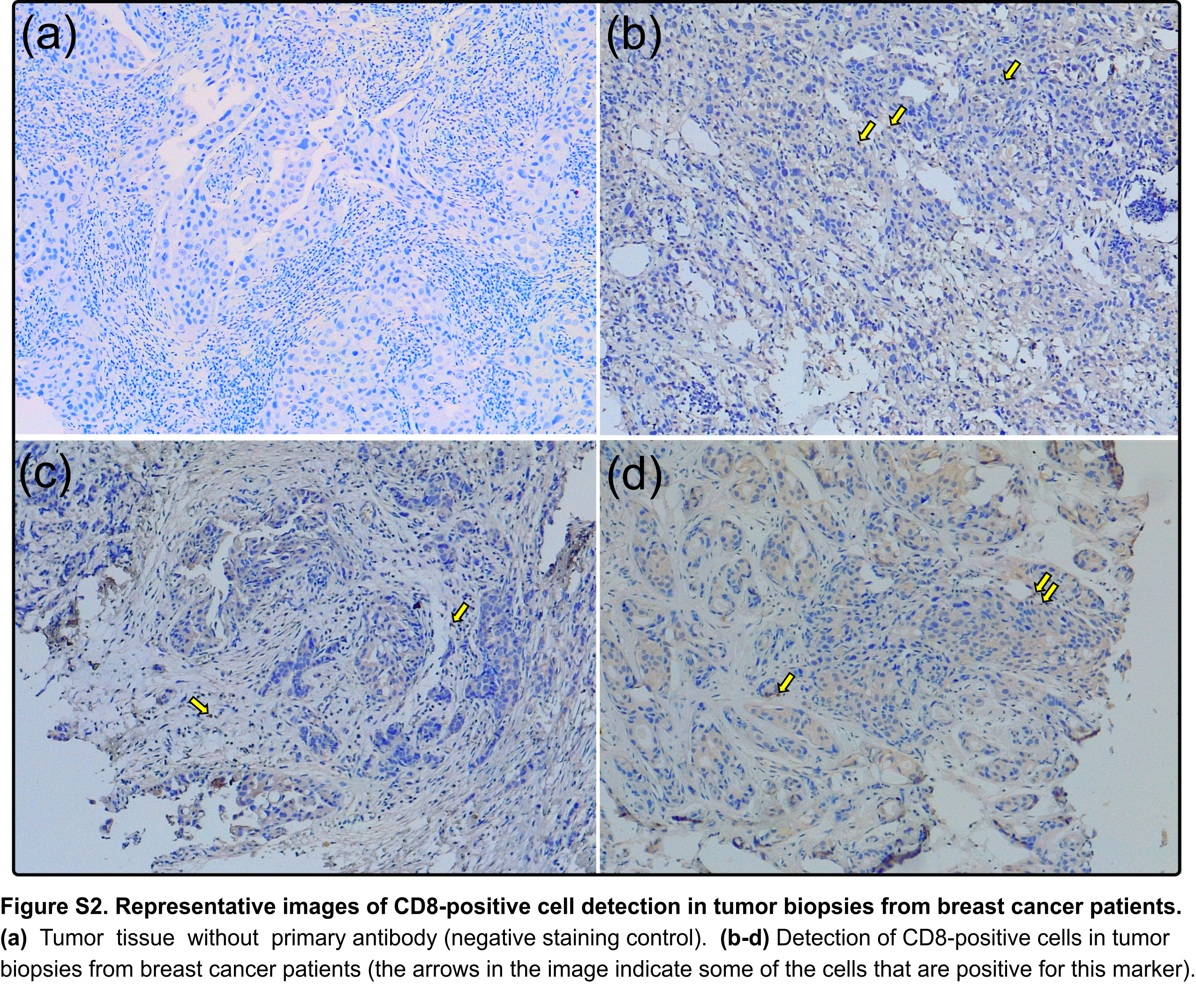

Supplement: Supplementary file 1 [file biomedicines-14-00663-s001.zip › Figure S2.tiff]

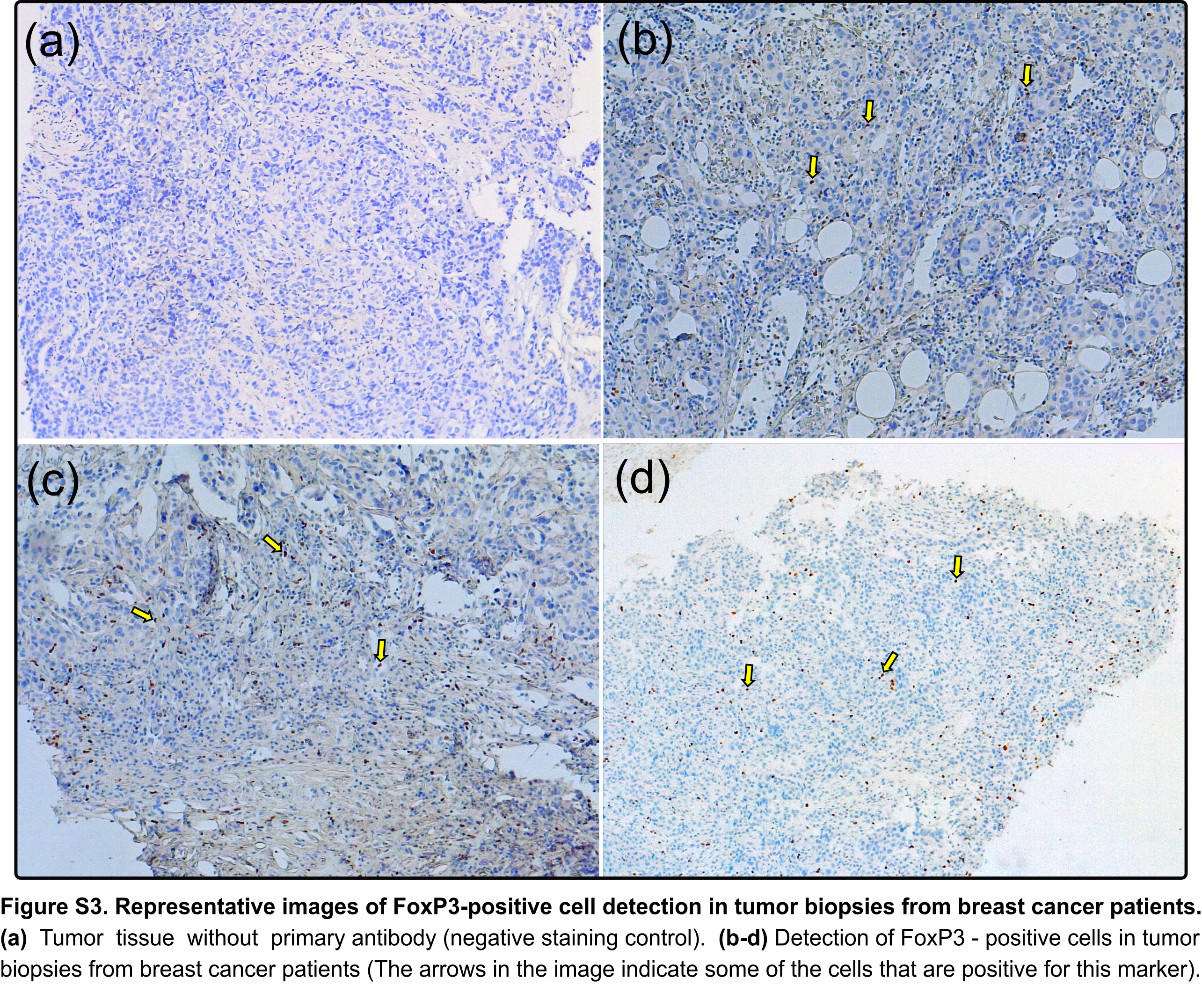

Supplement: Supplementary file 1 [file biomedicines-14-00663-s001.zip › Figure S3.tiff]

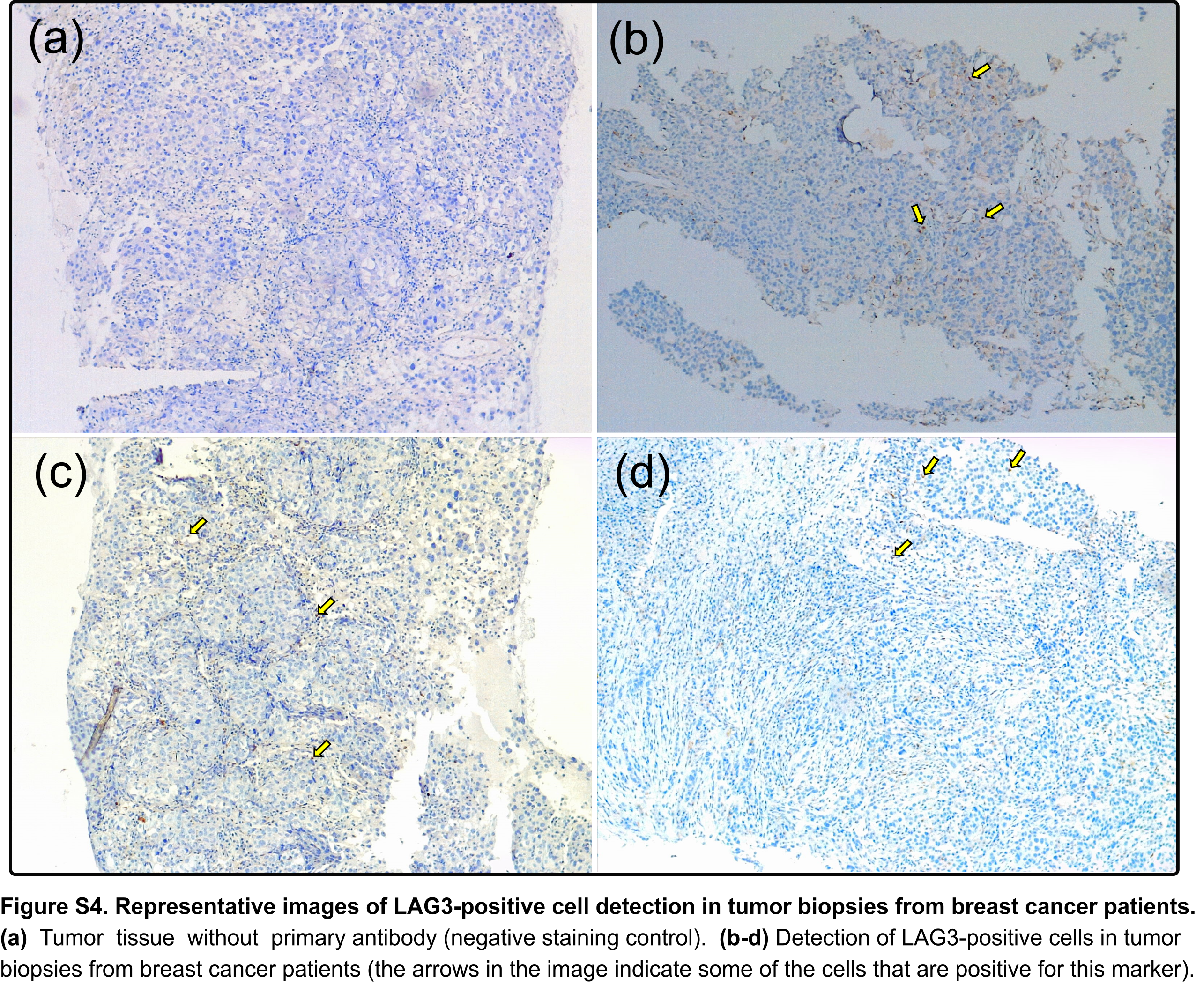

Supplement: Supplementary file 1 [file biomedicines-14-00663-s001.zip › Figure S4.tiff]

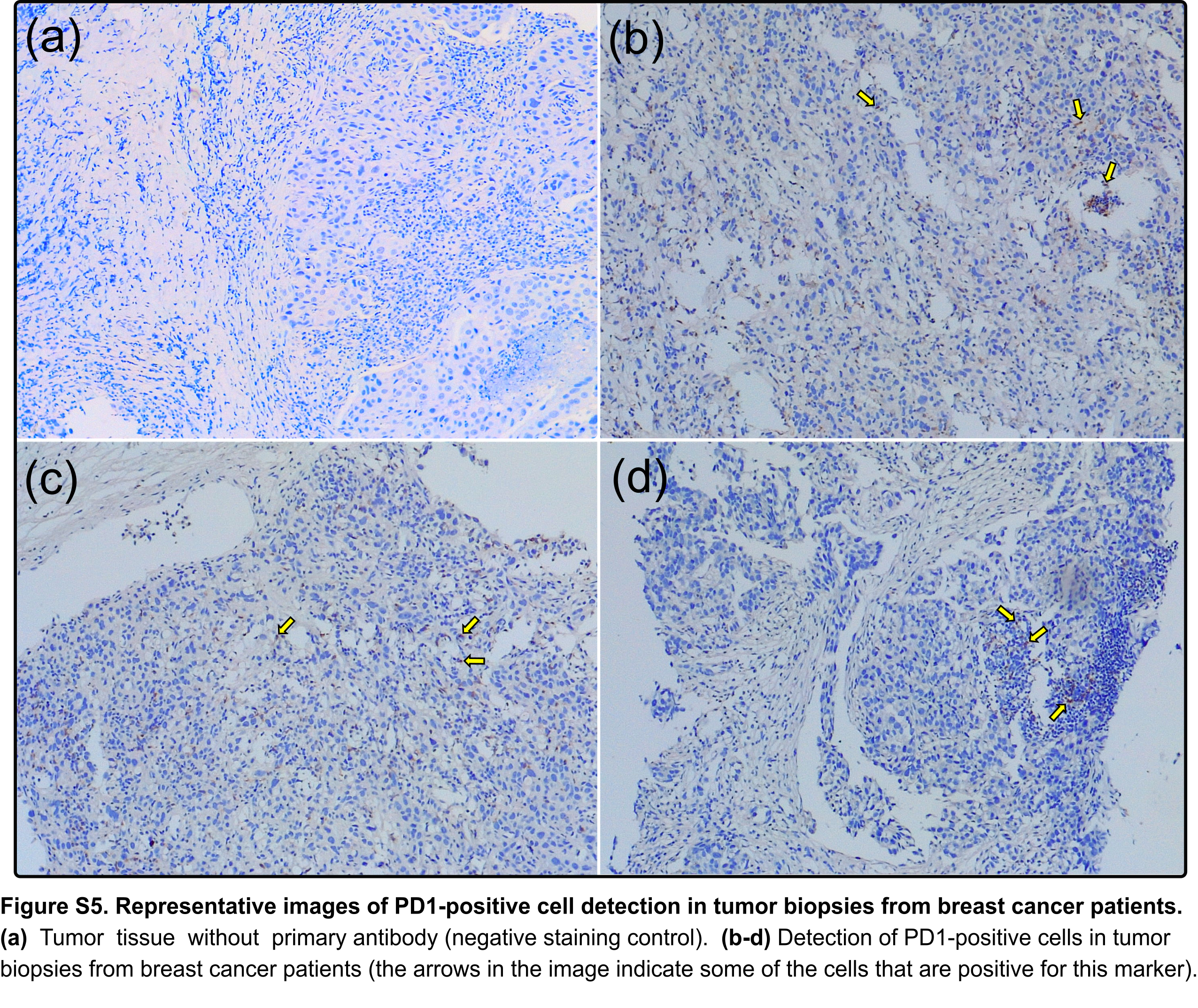

Supplement: Supplementary file 1 [file biomedicines-14-00663-s001.zip › Figure S5.tiff]

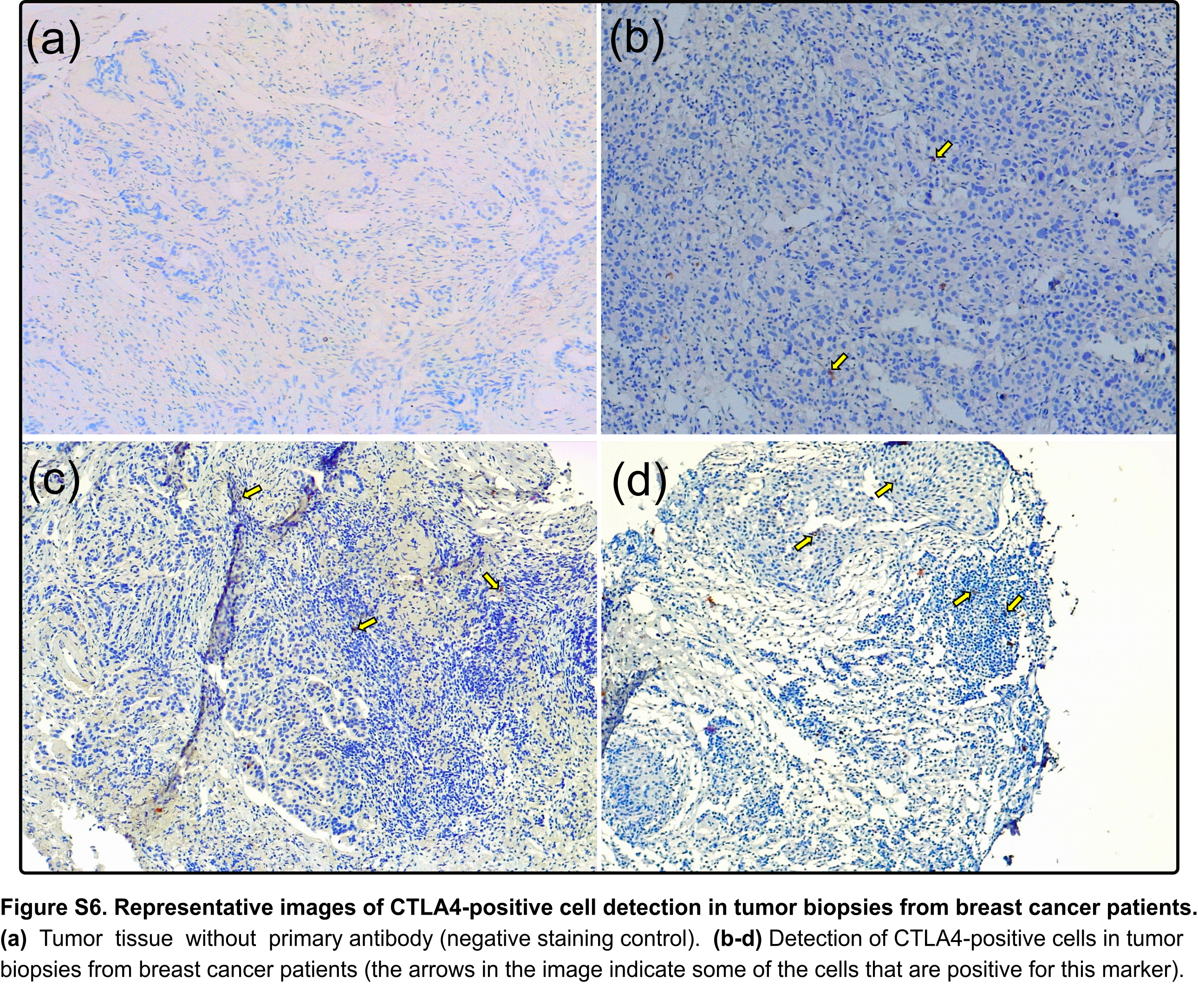

Supplement: Supplementary file 1 [file biomedicines-14-00663-s001.zip › Figure S6.tiff]

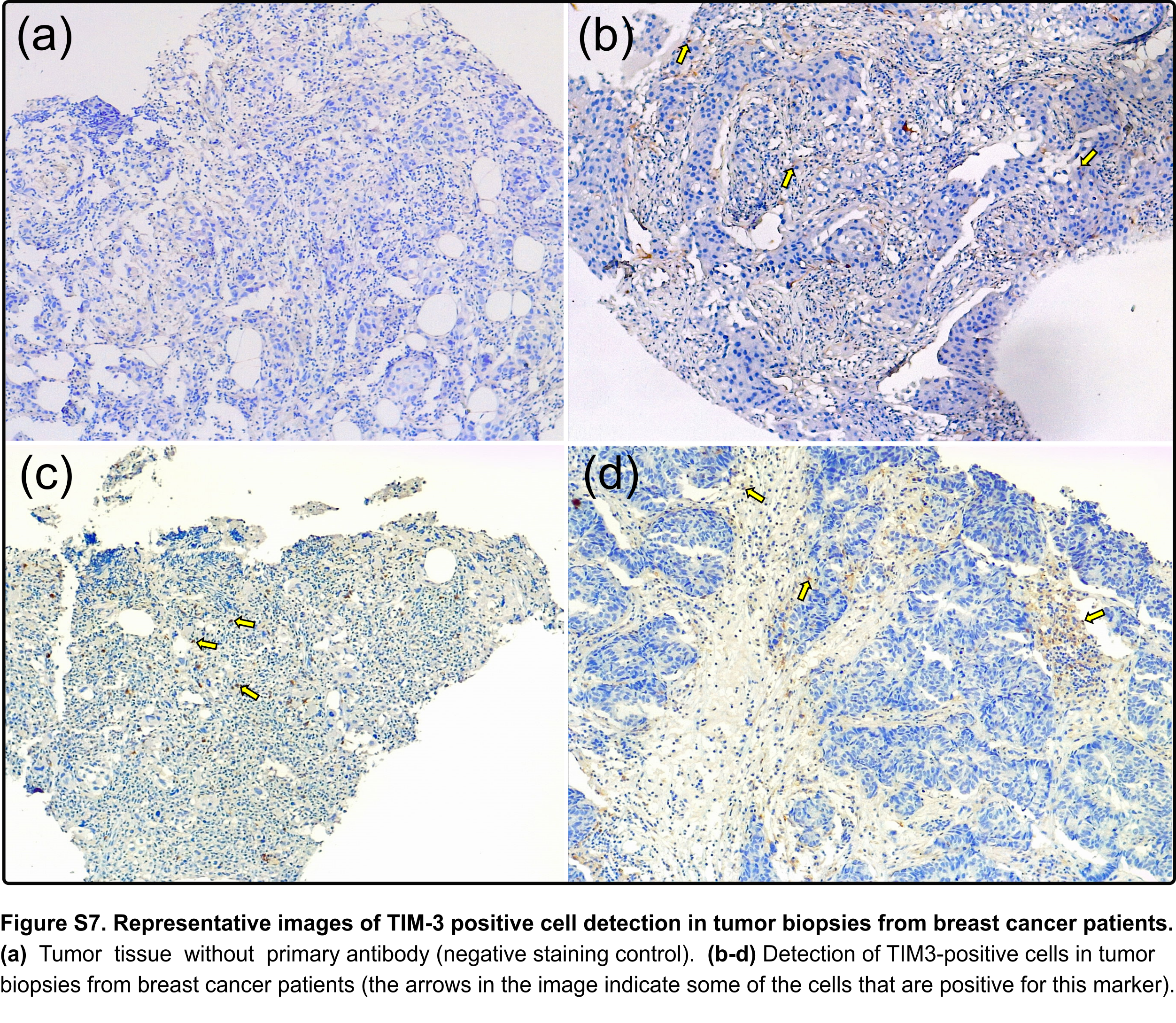

Supplement: Supplementary file 1 [file biomedicines-14-00663-s001.zip › Figure S7.tiff]

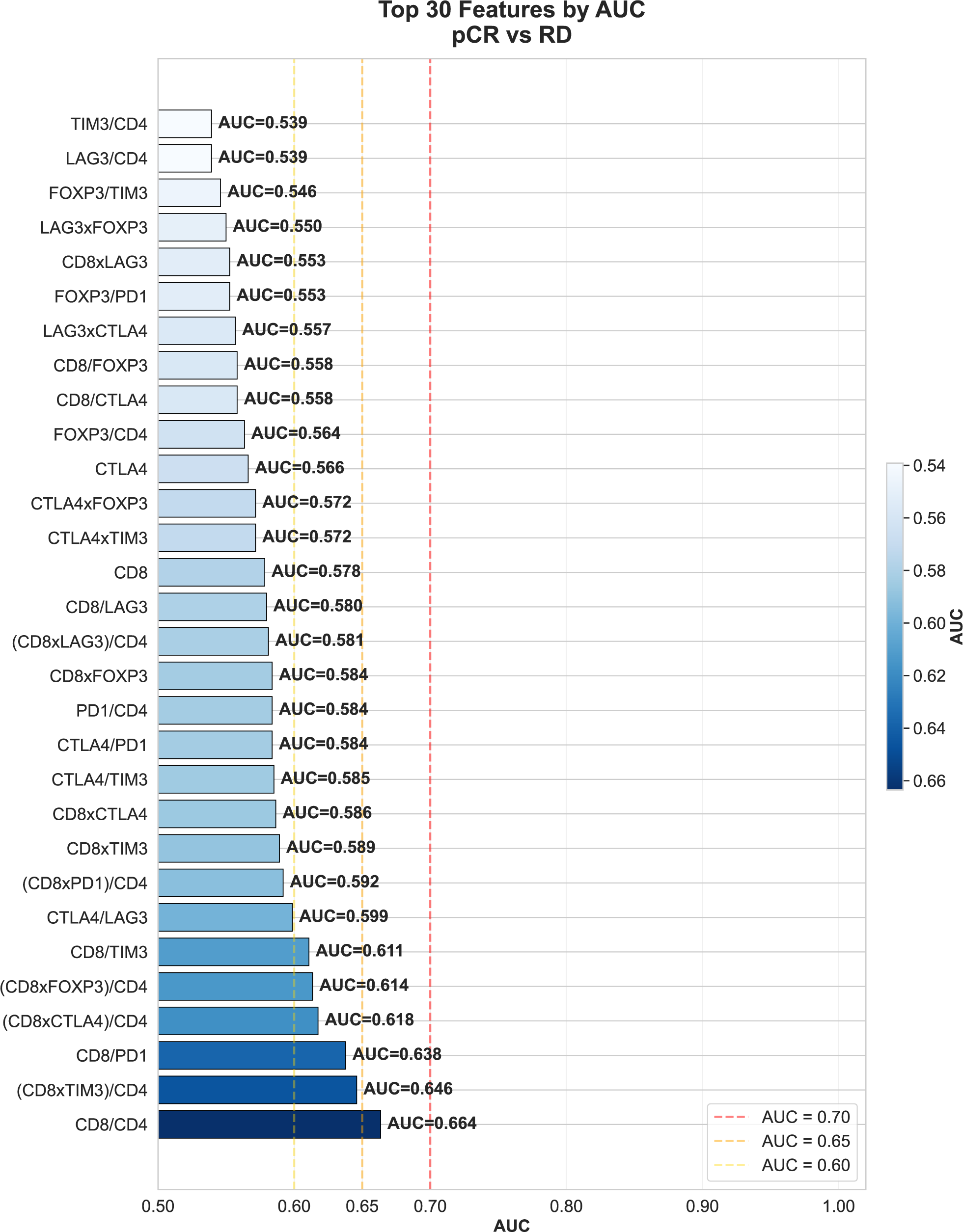

Supplement: Supplementary file 1 [file biomedicines-14-00663-s001.zip › Figure S8.tif]
